# Integrating Molecular Analysis and the Pharmacology Network to Discover the Antioxidative Effects of *Zanthoxylum piperitum* Fruits

**DOI:** 10.3390/plants15010148

**Published:** 2026-01-04

**Authors:** Ducdat Le, Thinhulinh Dang, Thientam Dinh, Soojung Yu, Vinhquang Truong, Minhee Kim, Su-Yun Lyu, Kwang Seok Ahn, Mina Lee

**Affiliations:** 1 College of Pharmacy and Research Institute of Life and Pharmaceutical Sciences, Sunchon National University, 255 Jungangno, Suncheon, Jeonnam 57922, Republic of Korea; ddle@scnu.ac.kr (D.L.); 1220173@s.scnu.ac.kr (T.D.); 1243010@s.scnu.ac.kr (T.D.); 1243011@s.scnu.ac.kr (V.T.); suyun@scnu.ac.kr (S.-Y.L.); 2 Department of Natural Cosmetics Science and Smart Beautytech Research Institute, Sunchon National University, 255 Jungangno, Suncheon, Jeonnam 57922, Republic of Korea; sjyu@scnu.ac.kr; 3 Department of Forest Landscape Architecture, Dongshin University, Naju, Jeonnam 58245, Republic of Korea; 4 Department of Science in Korean Medicine, Kyung Hee University, 24 Kyungheedae-ro, Dongdaemun-gu, Seoul 02447, Republic of Korea

**Keywords:** *Zanthoxylum piperitum*, UHPLC-MS/MS, LC-DPPH, antioxidant, network pharmacology, molecular docking

## Abstract

*Zanthoxylum piperitum* is a food and culinary plant commonly used in East Asia. In traditional medicine, its fruits, seeds, and bark have been utilized to treat digestive disorders, pain, and stomachache. Prior research has demonstrated its health benefits, particularly its significant antioxidant properties. However, limited research has investigated the specific metabolites responsible for these pharmacological effects. In this study, the antioxidant activities (EC_50_: 9.1–1084.5 μg/mL) and metabolite profiles of different organs (fruits, pericarps, and seeds) of *Z. piperitum* collected from different regions were comparatively analyzed. Chemical structures of 91 metabolites from different organs were identified using UHPLC-Orbitrap-MS/MS based on untargeted metabolomics. The LC-DPPH method was employed to screen antioxidants from the extracts of the most active organ (the pericarps). The potential effects of the active compounds on oxidation-related diseases were evaluated by integrating compound–target interaction network analysis. Protein–protein interaction (PPI) networks revealed EGFR, STAT3, AKT1, TNF, BCL2, CASP3, ESR1, PPARA, CYP19A1, and CDK2 as central hub genes. The significance of compound and target interactions was further supported by molecular docking studies, which demonstrated favorable binding affinities, with most proteins exhibiting docked scores below −4.27 kcal/mol. The extracts of *Z. piperitum* fruits and pericarps also exhibited antioxidative activity against ROS production in LPS-stimulated RAW264.7 cells. Our findings demonstrate the application of an optimized extraction process and underscore the medicinal value of this food-plant by characterizing its bioactive constituents. The results indicate that *Z. piperitum* may serve not only as a health-promoting food but also has the potential for prevention or treatment of oxidative-stress-related diseases. Future research should focus on in vivo studies by exploring the therapeutic mechanisms of actions of the active extracts.

## 1. Introduction

*Zanthoxylum piperitum* (L.) DC., also known as Korean or “초피,” is a deciduous, fragrant, and aromatic shrub belonging to the Rutaceae family. *Z. piperitum* is widely distributed throughout East Asia, especially in Korea, Japan, and China. This species has been traditionally utilized in medicine and culinary practices and is recognized for its abundance of bioactive natural products. Multiple parts of *Z. piperitum*, such as the pericarps, seeds, leaves, and stems, have long been used to address digestive disorders, toothache, and neuralgia, as well as for their tonic properties [1,2]. In addition, *Z. piperitum* pericarps are routinely consumed to enhance the flavor of meals. Several studies have reported the presence of secondary metabolites, including alkaloids, flavonoids, lignans, coumarins, terpenoids, and essential oils, found in the ethanolic extract of the aerials parts [3] and leaves [4], and in the methanolic extract of stems [5] of *Z. piperitum*. Sanshool-type alkylamides are notable constituents in this plant, imparting its distinctive flavor profile. These derivatives are also linked to the plant’s medicinal activities, including pain relief, anti-inflammatory effects, and gastric protection [3,6,7]. Essential oils derived from the pericarp are rich in monoterpenes like limonene and β-phellandrene, exhibiting significant antibacterial, antioxidant, and insecticidal activities [8]. The growing interest in functional foods and natural medicine has drawn further attention to *Z. piperitum* as a valuable source of bioactive compounds. Recent pharmacological research has substantiated several traditional applications of this plant by demonstrating a range of biological activities, including antioxidant, anti-obesity, anti-neurodegenerative, anticarcinogenic, and cardiovascular protective effects [9,10,11]. The evidence from both in vitro and in vivo studies underscores the therapeutic potential of *Z. piperitum* and its active constituents.

However, limited studies have explored the biological activities or systematically identified the bioactive constituents and their potential mechanisms in *Z. piperitum*. Advanced approaches such as metabolomics, network pharmacology, and molecular docking are increasingly applied to bridge this knowledge gap, thereby reducing both time and resource requirements in identifying key molecules and their potential targets. This study aims to provide an in-depth investigation of the phytochemical composition and biological functions of *Z. piperitum* using integrated analytical and computational strategies. Our focus lies in identifying active metabolites and elucidating their potential health benefits. Additionally, an optimized extraction strategy was established to obtain antioxidant-rich extracts with high efficiency.

## 2. Results

### 2.1. Screening Antioxidative Effects of Z. piperitum Extracts

Extraction was performed using ethanol–water solvent systems with ethanol concentrations of 0%, 20%, 40%, 60%, 80%, and 100%, applied to three plant organs (fruits, pericarps, and seeds) collected from two regions [Sangdong-myeon, GPS coordinates: 35.30324, 127.4247, (산동면, SDM) and Gurye-eup, GPS coordinates: 35.19979, 127.4096 (구례읍, GRE)]. As a result, 36 distinct extracts were successfully obtained, encompassing Sangdong-myeon fruits (SDM_F), pericarps (SDM_P), and seeds (SDM_S), together with Gurye-eup fruits (GRE_F), pericarps (GRE_P), and seeds (GRE_S), each prepared using ethanol–water solvent systems at concentrations of 0%, 20%, 40%, 60%, 80%, and 100% ethanol, and subsequently recovered following solvent removal under reduced pressure. These extracts were evaluated for their antioxidative properties by assessing their DPPH and ABTS radical scavenging activities. ABTS is a water-soluble radical that displays a distinct green-blue color in its oxidized state (ABTS^+•^). Upon treatment with *Z. piperitum* extracts, the ABTS solution color changed from green-blue to green or faded to colorless, depending on the concentration of the sample. Among the GRE samples, P extracted with 0–60% EtOH and F extracted with 40–80% EtOH demonstrated potent ABTS radical scavenging activity, with EC_50_ values ranging from 76.8 to 91.8 μg/mL relative to the other GRE extracts. Nearly all seed extracts and the F100 extract exhibited weak ABTS radical scavenging effects under the experimental conditions. For the SDM samples, fruits extracted with 20–100% EtOH and P extracts displayed markedly stronger antioxidative effects (EC_50_ values: 55.9–89.8 μg/mL) compared with the other SDM extracts. SDM seed extracts also showed limited ABTS radical scavenging activity under experimental conditions. In the DPPH assay, the original solution was characterized by a deep violet color due to the presence of DPPH^•^ radicals. This color intensity decreased or turned colorless depending on the reaction between the DPPH^•^ radicals and the applied samples. After exposure to *Z. piperitum* extracts, the color of the DPPH solution was diminished. Among the SDM extracts, the P extracts induced a pronounced reduction in the DPPH solution color, with EC_50_ values in the range of 48.0–80.1 μg/mL, followed by a moderate effect in the fruit extracts (EC_50_ values, 85.5 to 184.1 μg/mL). The seed extracts had limited activity against DPPH radical scavenging (EC_50_ values: 242.1–1011.7 μg/mL). GRE extracts also mediated the reduction in the DPPH solution color due to scavenging of DPPH^•^ radicals. Specifically, P extracts (EtOH: 0%, 20%, 40%, 60%) and F extracts (40% EtOH) exhibited robust DPPH radical scavenging activity, with EC_50_ values from 62.6 to 93.3 μg/mL, compared to the other organ extracts.

**Figure 1:**
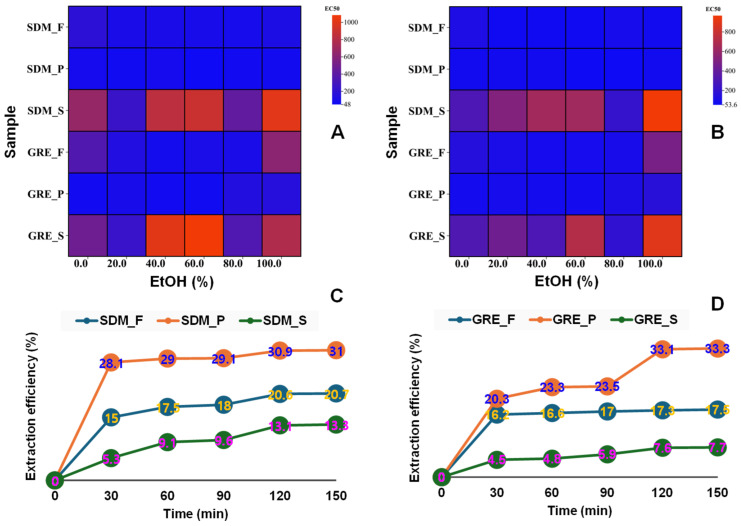
Heatmap analysis between antioxidant capacity and different extracts (**A**,**B**), and optimization of extraction time (**C**,**D**) of *Z. piperitum*.

The seed extracts demonstrated relatively low DPPH radical scavenging activity, with EC_50_ values exceeding 236.9 μg/mL (Table 1, Figure 1A,B).

Based on these data, it was evident that nearly all the P extracts exhibited higher antioxidant activity (indicated by lower EC_50_ values) compared to the fruit and seed extracts. Ethanol concentration plays a major role in determining antioxidant yield, with 40–60% EtOH producing some of the most potent extracts. Antioxidant activities of SDM under the extraction conditions surpassed those of the GRE. Among the fruit extracts, the SDM fruit extract (80% EtOH) demonstrated the highest activity (EC_50_ = 53.63 µg/mL) for ABTS radical scavenging, whereas the GRE fruit extract showed the lowest activity. In the GRE fruits, antioxidative activities increased from 147.4 (0% EtOH) to 81.8 (40% EtOH) before dropping sharply at 100% EtOH (EC_50_ = 480.5 µg/mL). Fruit extracts showed higher efficiency when extracted with ethanol of moderate polarity (40–60% EtOH), resulting in a greater concentration of antioxidant-rich compounds. The SDM pericarps exhibited the strongest activity (EC_50_ = 55.9 µg/mL) when extracted with 60% EtOH. The GRE_P extracts displayed greater variability and showed significant potential for ABTS radical scavenging activity in both 40% and 60% EtOH. The seed extracts from all *Z. piperitum* organs consistently showed weak antioxidative activity for ABTS radical scavenging under the same experimental parameters. To investigate the association between extract solvent systems and the radical scavenging activity of *Z. piperitum* organs, it was observed that the most effective extraction range, 40–60% EtOH, correlated with the lowest EC_50_ values, particularly for P extracts (Figure 1B). When 0% and 100% EtOH were utilized as solvents, antioxidant activity generally declined, likely due to the inefficient extraction of non-polar and polar antioxidant compounds, respectively.

The antioxidative capacities of the active organ (fruit and pericarps) extracts were further evaluated for their inhibitory effects on reactive oxygen species (ROS) production in cells [12]. The data represented the concentration needed to inhibit 50% of ROS formation. Both fruit and pericarp extracts showed ethanol concentration-dependent antioxidant activity. Notably, extracts with a higher ethanol content demonstrated considerably increased effectiveness. The IC_50_ values for the fruit extracts declined from 729.4 to 38.0 µg/mL for SDM and from 471.9 to 34.4 µg/mL for GRE as the ethanol concentration increased from 0% to 100%. Similarly, the pericarp extracts showed a strong trend, with IC_50_ values falling from 102.3 to 17.9 µg/mL for SDM and from 80.1 to 9.1 µg/mL for GRE. Among all samples tested, the 100% ethanol extract of the GRE pericarp exhibited the most potent activity (IC_50_ = 9.1 µg/mL), surpassing even the positive control (ascorbic acid, IC_50_ = 10.6 µg/mL). These results indicate that pericarp extracts, particularly those from GRE and extracted with 100% ethanol, contain highly effective antioxidative compounds capable of suppressing ROS production (Table 2). All tested samples did not show any significant effect on cell viability under experimental conditions (Figure S1, Supplementary Materials).

### 2.2. Optimization of Extraction Time

To assess extraction time, fruits, pericarps, and seeds of *Z. piperitum* collected from SDM and GRE locations were extracted for the specified durations of 0, 30, 60, 90, 120, and 150 min using different EtOH ratios in water. For the SDM samples, the extraction efficiency of the fruits and pericarps increased primarily within the first 30 min (28.1%), then showed only a marginal rise (28.1–30.9%) between 30 and 120 min, indicating a high but quickly plateauing extraction. The extraction efficiency remained relatively stable, approximately 13.3% at 150 min. Consequently, an incubation period of 120 min was chosen for all subsequent experiments (Figure 1C). In contrast, the extraction yield trend for the GRE pericarps differed from that of the fruits and seeds over the same time frames (Figure 1D). Specifically, the pericarp extracts from the GRE samples exhibited more pronounced changes in extraction efficiency compared to that of the other organs, starting at 20.3% at 30 min and showing a marked increase from 23.5% to 33.1% between 90 and 120 min. The P extract efficiency reached 33.3% in 150 min. Conversely, GRE_F showed comparatively modest and consistent extraction efficiency, ranging from 17.5% at 150 min with minimal variation after 60 min, suggesting rapid saturation or stabilization of extraction. GRE_S displayed the lowest extraction efficiency overall, with only a slight increase from 4.5% at 30 min to 7.7% at 150 min, indicating limited extractability under the experimental conditions. Notable variations in extraction efficiency were observed, highlighting the influence of the extraction matrix or method on compound recovery and identifying the GRE pericarps as the most efficient among the groups. Therefore, the optimal extraction time was determined to be 120 min for both pericarp and seed extraction and 60 min for fruit extracts. These findings carry noteworthy implications for optimizing extraction procedures aimed at specific compound profiles. The final extraction solvent and time used were based on the procedure described above. Comprehensive extraction efficiency data are provided in Table 3 and Table S1 (Supplementary Materials).

### 2.3. Identification of Active Components by Application of LC-DPPH

Pericarp extracts from SDM and GRE exhibited high antioxidative activity. Consequently, we employed an LC-DPPH method [13] to investigate the active compounds within these extracts. For this investigation, an LC-DPPH system was established to detect and characterize the active compounds by selecting peaks that demonstrated variable detection between DPPH-treated and untreated samples. This method facilitates large-scale screening for active ingredients (Table S2, Supplementary Materials). In the SDM_P extract, peak 15 (7-hydroxy-coumarin, *t*_R_ 7.585 min, *m*/*z* 163.0380) demonstrated the greatest reduction of 39.8% in peak detection between treated and non-DPPH treated extracts, followed by peak 13 (cryptochlorogenic acid, *t*_R_ 7.571 min, *m*/*z* 353.0865, 35.2%), peak 14 (*t*_R_ 7.566 min, *m*/*z* 375.1969, 32.6%), peak 10 (*t*_R_ 7.470 min, *m*/*z* 597.1787, 22.9%), peak 23 (rutin, *t*_R_ 12.164 min, *m*/*z* 611.1582, 22.8%), peak 24 (myricetin-3-*O*-pentoside, *t*_R_ 12.175 min, *m*/*z* 449.0710, 19.5%), and peak 9 (chlorogenic acid, *t*_R_ 7.370 min, *m*/*z* 353.0865, 18.8%). The chemical structures of these peaks were identified through comprehensive analysis of precursor ions, isotope distribution, and fragmentation pathways of their mass spectra, together with spectral comparison using mass databases (GNPS, HMDB, American mass bank) and an in-house database. Among these, peaks 9 (*t*_R_ 7.370 min, *m*/*z* 353.0865), 14 (*t*_R_ 7.571 min, *m*/*z* 353.0865), and 23 (*t*_R_ 12.164 min, *m*/*z* 611.1582) were confirmed using reference standards. The remaining active compounds were identified using the GNPS-FBMN online tool and MS DIAL (version 5.1.230912) software in conjunction with mass databases. These included three flavone glycosides, two quinic acids, one coumarin, and one phenolic glycoside (Figure S2 and Table S3, Supplementary Materials). The GRE_P extract revealed eight active compounds, including peaks 27 (*t*_R_ 9.301 min, *m*/*z* 374.1578, 29.9%), 29 (*t*_R_ 9.880 min, *m*/*z* 482.2327, 27.1%), 13 (*t*_R_ 6.478 min, *m*/*z* 182.1165, 28.4%), 61 (*t*_R_ 20.16 min, *m*/*z* 184.1319, 26.2%), 46 (*t*_R_ 16.934 min, *m*/*z* 335.2168, 24.0%), 17 (*t*_R_ 7.138 min, *m*/*z* 190.0488, 20.5%), 39 (*t*_R_ 13.158 min, *m*/*z* 302.1271, 19.3%), and 18 (*t*_R_ 7.143 min, *m*/*z* 191.0196, 17.9%). Structural analysis indicated these were composed of two alkaloids, two carboxylic acids, one phenol, one terpenoid, and one unknown compound (Figure S3 and Table S4, Supplementary Materials).

### 2.4. Antioxidant Diseases Investigated Through Network Pharmacology

**Figure 2:**
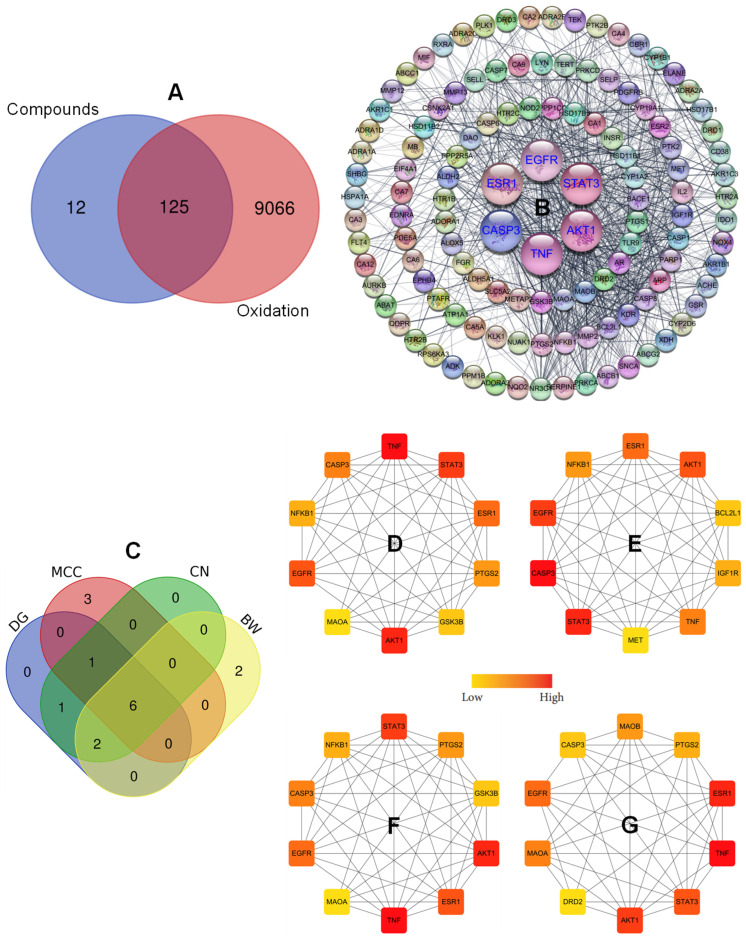
Intersecting targets between GRE-metabolite and oxidation-related targets identified by using the Venn diagram (**A**). Full PPI network (**B**). The Venn diagram (**C**) was used to identify top-ranked nodes based on degree ((**D**): DG), maximal clique centrality ((**E**): MCC), closeness ((**F**): CN), and betweenness ((**G**): BW). Color intensity of nodes represents the high score of centralities.

Network pharmacology is an emerging interdisciplinary field grounded in systems biology, bioinformatics, and network theory, which provides a robust approach for analyzing the complex and interconnected relationships among drugs, targets, and diseases. In contrast to traditional pharmacology which typically focuses on single-target and single-drug interactions, network pharmacology introduces the concept of multi-target therapeutics, thereby aligning with a systems-level perspective of biological processes [14]. Through this approach, researchers are able to construct drug–target–disease networks, identify synergistic effects, and predict potential off-target activity. This strategy yields valuable insights into mechanisms of action, toxicity prediction, and the repurposing of bioactive compounds. In this study, the application of network pharmacology to the formula enabled the identification of potential antioxidant-related targets for oxidative stress disorders, resulting in 9191 targets associated with antioxidation identified from the GeneCards (threshold score > 5.0) and DisGeNET databases. Following integration and removal of duplicate entries, 147 unique targets of the seven active compounds from the SDM pericarp extract were obtained. Overlap analysis using a Venn diagram identified 125 intersecting targets between component- and oxidation-related targets (Figure 2A). These intersecting targets were imported into the STRING web tools to construct a protein–protein interaction (PPI) network by setting *Homo sapiens* as the species. The output result was transferred into the Cytoscape software (Ver. 10.1) using the STRING plug-in, to visualize the interaction network of overlapping targets (Figure 2B). The resulting network comprised 122 nodes (non-connected nodes were excluded) and 963 edges representing action targets. Then, the Cytohubba algorithm was applied to identify the critical targets by analyzing the central scores such as degree (DG), maximal clique centrality (MCC), closeness (CN), and betweenness (BW), which were analyzed. The Venn diagram was applied to identify crucial genes (Figure 2C). As a result, six hub genes were obtained, AKT1, TNF, STAT3, EGFR, ESR1, and CASP3, as potential targets against oxidation-related diseases.

**Figure 3:**
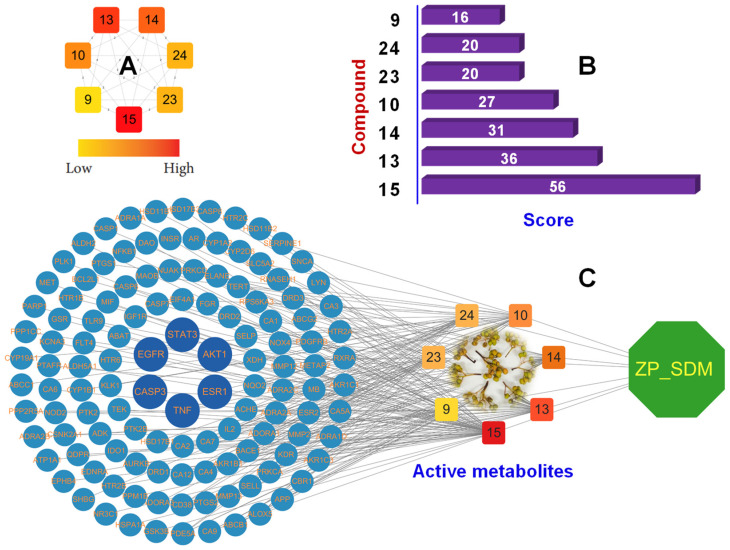
Integrated network pharmacology analysis of *Z. piperitum* collected in the SDM region (ZP_SDM). (**A**) Degree values of the seven key active metabolites (orange rectangle nodes), with colors indicating low to high connectivity. (**B**) Top-ranking active compounds sorted by central score. (**C**) Compound–target network of *Z. piperitum*: targets are represented as blue circle nodes highlighting core targets (STAT3, EGFR, CASP3, TNF, ESR1, and AKT1) and their interactions with the active metabolites. The plant is a green octagon node with the yellow label “ZP_SDM”.

Subsequently, an SDM-metabolite–target network was established to investigate the associations between active metabolites and antioxidant-related targets. This analysis revealed multiple connections between SDM metabolites and antioxidant targets, suggesting compound synergies within the network pharmacology framework. In this network pharmacology analysis, ligands and proteins are illustrated as round brown rectangles and pink ellipse nodes, respectively. The active metabolites were listed based on their connectivity values of 56, 36, 31, 27, 20, 20, and 16 for peaks 15, 13, 14, 10, 23, 24, and 9, respectively (Figure 3A,B). These findings may indicate that the antioxidative potential of these compounds is mediated through modulation of oxidation-related targets. Finally, a comprehensive metabolite–protein–target interaction network was developed, demonstrating the connections between *Z. piperitum* metabolites from the SDM_P extract and oxidation-related targets within the pharmacology network (Figure 3C), thereby providing a systematic overview of each metabolite’s interactions with multiple targets.

Similarly, for the selected compounds in GRE, associated targets were identified using SwissTargetPredict (https://www.swisstargetprediction.ch/, accessed on 2 June 2025), resulting in 210 targets corresponding to seven metabolites after duplicate targets were excluded.

**Figure 4:**
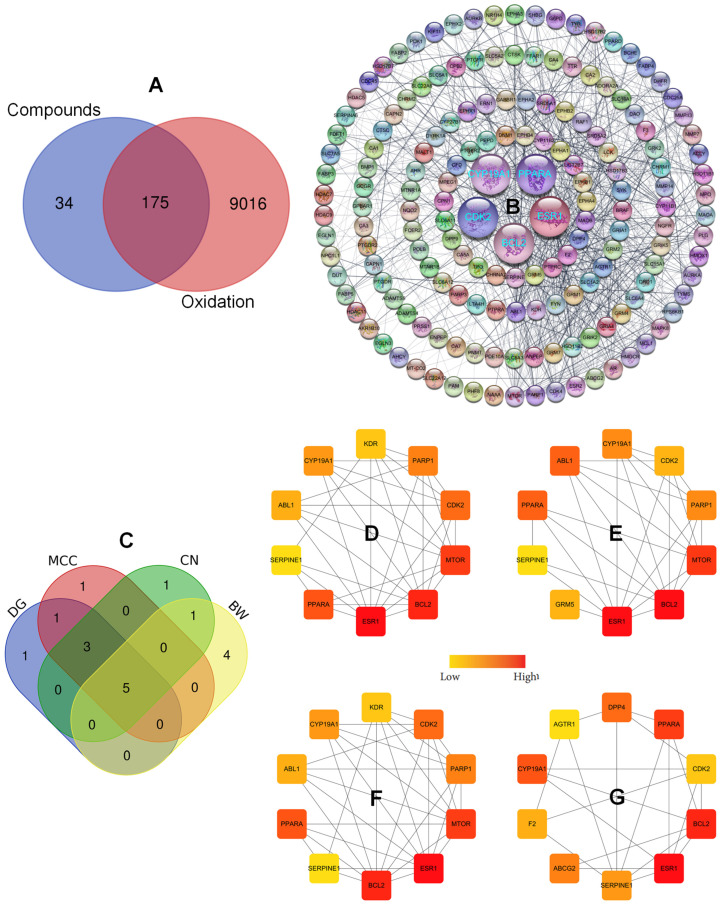
Intersecting targets between SDM-metabolite and oxidation-related targets identified by using the Venn diagram (**A**). Full PPI network (**B**). The Venn diagram (**C**) was used to identify top-ranked nodes based on degree ((**D**): DG), maximal clique centrality ((**E**): MCC), closeness ((**F**): CN), and betweenness ((**G**): BW). Color intensity of nodes represents the high score of centralities.

**Figure 5:**
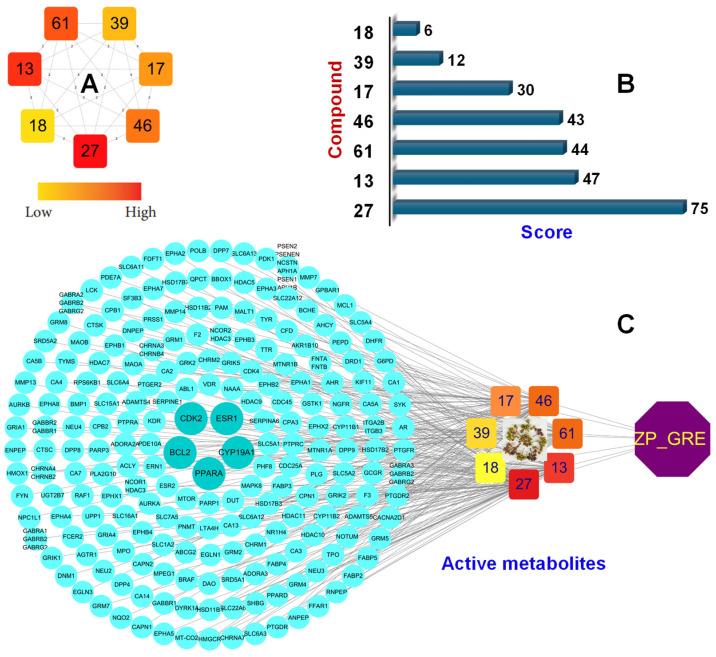
Integrated network pharmacology analysis of *Z. piperitum* collected in the GRE region (ZP_GRE). (**A**) Degree distribution of seven key active metabolites, with colors indicating low to high connectivity. (**B**) Top-ranking candidate compounds ordered by network score. (**C**) Compound–target network of *Z. piperitum*: targets are represented as blue circle nodes highlighting core targets (CDK2, BCL2, PPARA, CYP19A1, and ESR1) and their interactions with the active metabolites. The plant is a purple octagon node with the yellow label “ZP_GRE”.

Next, 176 overlapping oxidation-related targets were determined for creating the PPI network (Figure 4A), which was visualized by using the Cytoscape software. The core targets were subsequently determined due to high central scores (DG, CN, MCC, and BWW) by using the Cytohubba plug-in in the Cytoscape, followed by the Venn diagram, to find the intersecting targets based on the central scores (Figure 4B). Thus, five hub genes, ESR1, PPARA, BCL2, CYP19A1, and CDK2, are considered as important targets against oxidation-related diseases (Figure 4C). A metabolite–target interaction network was established showing interactions of compounds and targets. The metabolites were further categorized in descending order based on degree values determined by the Cytohubba plug-in in the Cytoscape network, specifically comprising 27 (score 75), 13 (score 47), 61 (score 44), 46 (score 43), 17 (score 30), 39 (score 12), and 18 (score 6) (Figure 5A,B). Ultimately, a metabolite–oxidation target network was constructed to demonstrate multiple interactions between active constituents of *Z. piperitum* and oxidation-related targets (Figure 5C).

### 2.5. Molecular Docking Verification

**Figure 6:**
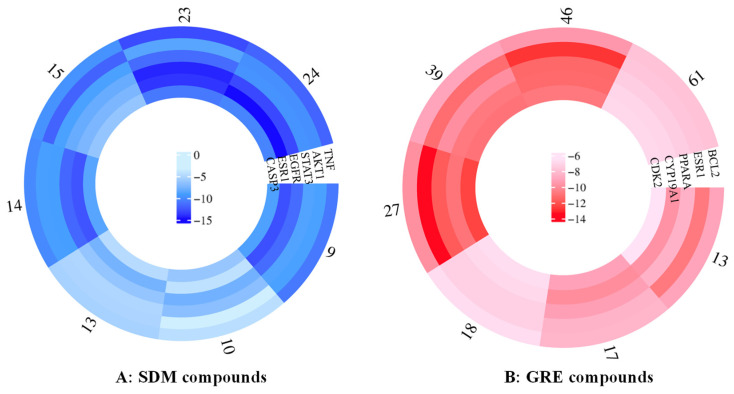
Circular heatmap of docked scores of the active compounds in *Z. piperitum* against oxidation collected from SDM ((**A**), blue color) and GRE ((**B**), red) locations. Color intensity signifies a robust binding affinity.

To explore the antioxidative potential of active compounds in *Z. piperitum*, the binding affinities between these compounds and core targets were evaluated by molecular docking. A heatmap was used to visualize the docking functional scores of each configuration (Figure 6). For the SDM compounds, compounds **9**, **14**, **23**, and **24** exhibited strong binding to TNF, EGFR, and ESR1 (ΔG: −14.81 to −10.91 kcal/mol). Additionally, compounds **23** and **24** showed notable affinity for STAT3 and CASP3 (ΔG: −11.57 to −9.74 kcal/mol). Moderate binding to AKT1 was observed for compounds **9**, **14**, **23**, and **24** (ΔG: −9.86 to −8.61 kcal/mol). Compound **10** displayed binding affinities towards STAT3, EGFR, and CASP3 (ΔG: −7.73 to −6.17 kcal/mol), while compounds **13** and **15** interacted with TNF, EGFR, and ESR1 (ΔG: −8.09 to −5.39 kcal/mol) (Figure 6A).

Similarly, for the GRE compounds, compounds **13**, **27**, **39**, and **46** consistently showed the lowest binding energies, indicating a strong predicted affinity toward all assessed targets. Particularly, compound **13** showed binding affinities toward ESR1 and CDK3 with noticeable binding scores of −10.72 and −11.55, respectively. Compound **46** displayed low docked scores against ESR1 (ΔG: −12.81 kcal/mol), CYP19A1 (ΔG: −11.27 kcal/mol), and PPARA (ΔG: −11.34 kcal/mol). On the other hand, compound **27** had the strongest binding to CDK2 (ΔG: −12.3 kcal/mol) and ESR1 (ΔG: −13.39 kcal/mol). In contrast, compounds **13** and **18** displayed comparatively similar interactions, with binding scores shifting to approximately −5.55 to −7.25 kcal/mol across most targets (Figure 6B).

### 2.6. Identification of Compounds from the Different Organ Extracts of Z. piperitum

**Figure 7:**
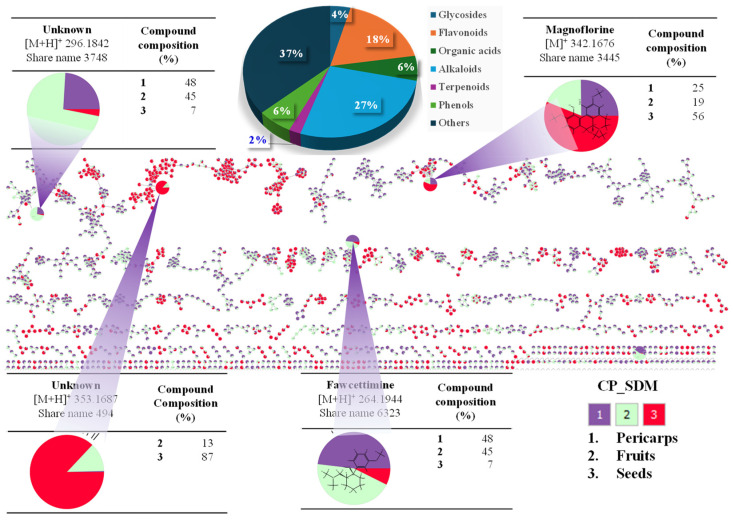
Feature-based molecular network-guided identification of highlighted metabolites distributed from the pericarp (1, purple color), fruit (2, mint color), and seed (3, red color) extracts of *Z. piperitum* collected in the SDM region.

**Figure 8:**
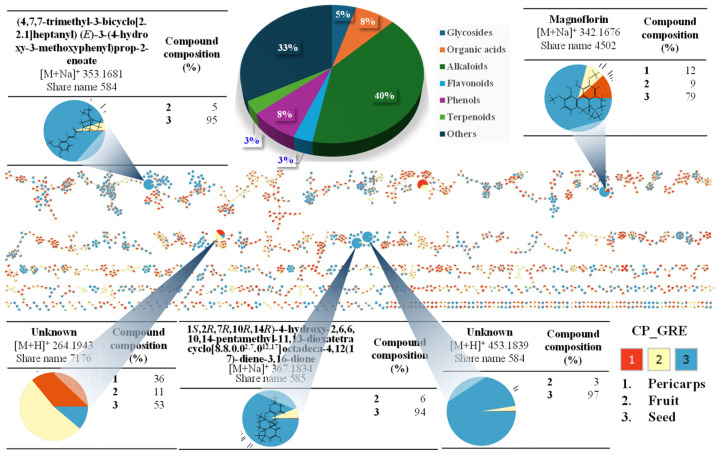
Feature-based molecular network-guided identification of highlighted metabolites distributed from the pericarps (1, purple color), fruits (2, mint color), and seeds (3, red color) collected in the GRE region.

The compounds were identified through the integration of positive and negative ion mode peak data, which were obtained and analyzed using the UHPLC-Orbitrap-MS/MS untargeted metabolomics technique (Figure 7 and Figure 8). Accordingly, 49 distinct compounds were identified in the pericarps, fruits, and seeds of *Z. piperitum* collected from the SDM region.

Among the compound groups, the alkaloid compound group is the largest proportion (27%) compared to other identified compounds, followed by groups such as flavonoids (18%), organic acids (6%), phenols (6%), glycosides (4%), terpenoids (2%), and other compounds present in smaller proportions. In contrast, the GRE samples yielded 64 identified compounds, predominantly alkaloids (40%), organic acids (8%), and phenols (8%), followed by glycosides (5%), flavonoids (3%), terpenoids (3%), and other minor groups (Figure 8). Details regarding the characteristics of the identified compounds are listed in Tables S3 and S4 (Supplementary Materials).

### 2.7. Relative Quantification of Compounds from Z. piperitum Extracts

The compounds identified in the *Z. piperitum* extracts were assessed for their relative concentrations based on the chromatogram analysis of the peak area ratio between the internal standard and the analyte. The contents of the target peaks were quantitated in relation to the internal standard being examined (Figure S11, Supplementary Materials). Among compounds identified from the GRE region, compounds **2**, **13**–**15**, **17**–**22**, **36**, **37**, and **61** exhibited concentrations relative to the internal standard of 12.51 ± 0.01, 5.87 ± 0.06, 5.09 ± 0.01, 5.56 ± 0.02, 7.99 ± 0.02, 7.85 ± 0.01, 7.64 ± 0.00, 8.46 ± 0.02, 8.31 ± 0.00, 8.26 ± 0.00, 10.57 ± 0.01, 10.36 ± 0.01, and 8.54 ± 0.01 μg/mL, respectively. Other compounds showed lower quantities (Figure S12 and Table S5, Supplementary Materials). Similarly, the compounds identified from the SDM region also displayed their contents. Compounds **2**, **3**, **6**, **13**, **15**, **18**, **21**, **23**–**31**, **41**, **42**, and **49** displayed concentrations relative to the internal standard of 9.23 ± 0.02, 6.99 ± 0.01, 9.20 ± 0.00, 9.60 ± 0.01, 9.03 ± 0.22, 9.03 ± 0.01, 10.07 ± 0.01, 15.46 ± 0.05, 15.07 ± 0.03, 13.75 ± 0.25, 5.28 ± 0.01, 13.60 ± 0.02, 13.72 ± 0.01, 12.16 ± 0.01, 5.21 ± 0.00, 5.44 ± 0.01, 8.53 ± 0.01, 7.48 ± 0.01, and 7.74 ± 0.01 μg/mL, respectively. Other compounds showed minimal quantities found in the extracts (Figure S13 and Table S6, Supplementary Materials).

## 3. Discussion

Extraction is a common technique for separating small molecules from plants. The quantity and diversity of compounds obtained depend on the extraction conditions as well as the physical parameters of the experiment. Therefore, optimizing extraction methodologies is essential for the isolation and identification of biomolecules from natural sources. In this study, an unconventional approach, ultrasonication-assisted extraction, was employed to obtain active compounds from multiple plant organs, including the fruits and pericarps of *Z. piperitum* collected from two regions in Korea. Extracts from the pericarps (notably, the SDM samples) demonstrated significant potential as antioxidants based on ABTS radical scavenging activity. The choice of extraction solvent polarity is a key factor influencing the induction of antioxidant capacity. Specifically, the mid-polar solvent mixture (40–60% EtOH) exhibited maximal extraction efficiency. The comparatively low antioxidant activity observed in the seed extracts from the two regions indicates the need to prioritize fruit and pericarp materials for future investigations. The pericarps of *Z. piperitum* (especially those extracted using 40–60% ethanol) consistently displayed superior ABTS radical scavenging efficacy across all tested samples. These results underscore the significance of both extraction parameters and plant tissue selection in maximizing antioxidant outcomes. Further bioactivity-guided fractionation and comprehensive phytochemical analysis of these extracts are warranted to pinpoint the compounds responsible for the observed bioactivity. Evaluation of DPPH radical scavenging further revealed that all crust extracts exhibited higher antioxidant activities, as evidenced by lower EC_50_ values across the tested EtOH concentrations and collection sites. In particular, P extracts derived from the SDM region achieved low EC_50_ values approaching or below 50 µg/mL at 0–60% EtOH concentrations, indicating a substantial abundance of highly active antioxidant constituents in these extracts.

Furthermore, the S extracts demonstrated the lowest antioxidant efficacy, as indicated by their high EC_50_ values, particularly in media with higher EtOH content. The highest recorded EC_50_ value was 1084.5 µg/mL for the SDM_S extract derived from 60% EtOH. These findings suggest that *Z. piperitum* seeds possess a moderate level of DPPH-reactive antioxidant compounds, which may be attributable to the relatively low concentrations of phenolic and/or flavonoid compounds present in this portion of the plant [15]. The fruit extracts generally showed moderate antioxidant activity, with the GRE fruit extracts displaying the weakest efficacy, particularly at 0% and 100% EtOH (EC_50_ > 300 µg/mL and 600 µg/mL, respectively). Moreover, the P extracts obtained via solvent extraction at 20–60% EtOH exhibited moderate to good DPPH radical scavenging activity, whereas the P extracts from SDM (0% and 100% EtOH) also demonstrated measurable DPPH radical scavenging capacity. These findings indicate efficient extraction of both hydrophilic and moderately lipophilic antioxidants [16]. Seed extracts were found to exhibit lower inhibition, making them less effective antioxidant sources. Accordingly, the optimal solvent extraction was determined to be 60% EtOH in water for isolating compounds from *Z. piperitum* organs. Both the ABTS and DPPH assays revealed comparable patterns across solvents and extract types. However, the ABTS radical scavenging activity tended to be lower and more consistent across concentrations in the active extracts, particularly those from the fruit, whereas DPPH exhibited a wider dynamic range. This variation can be attributed to the distinct chemical properties of ABTS and DPPH radicals (with ABTS being more susceptible to hydrophilic antioxidants and DPPH to lipophilic antioxidants). Thus, employing both assays provides a more comprehensive assessment of antioxidant capacity. These extracts have potential as promising agents for the treatment or prevention of oxidative-stress-related disorders. Further investigation is warranted to isolate and identify the principal bioactive compounds responsible for the observed activities in the extracts. In addition, research into synergistic interactions among metabolites and the optimization of solvent extraction procedures could improve extract efficacy. Additionally, the extracts of the fruits and pericarps were further evaluated for their antioxidative capacities toward ROS production induced in RAW264.7 cells. All the P extracts demonstrated higher antioxidative effects than those of the F extracts. Except for P (GRE, 40% and 60% ethanol), nearly all extracts displayed enhanced antioxidative activity in proportion to ethanol concentration. These data highlight the potential application of these extracts as natural antioxidant agents for managing oxidative stress responses. The antioxidative effects identified in this study have enhanced the therapeutic understanding of this food plant, recognized for its putative anti-inflammatory, anti-thrombogenic, and anticancer properties reported in previous reports [17,18,19]. Our results also emphasize this food material’s potential for creating skin care benefits [20] as well as enhancing health benefits, given its everyday use as a food flavoring. The effects on antioxidant capacity of various parts of the *Z. piperitum* have yielded substantial evidence for the selection of specific *Z. piperitum* organs from plants in the SDM and GRE regions as input materials for the development of product-rich antioxidants derived from local SDM and GRE sources. This research highlights the importance of utilizing native plant resources to enhance the nutritional profile of antioxidant products. Furthermore, it opens avenues for sustainable agricultural practices that can benefit both local economies and health.

The time-based extraction protocols were established to identify the optimal extraction time values. The protocols also assessed the extraction efficiencies of different organs (F, P, S) retrieved from SDM and GRE using varying EtOH concentrations to determine the most effective extraction conditions. For the F samples, the highest extraction efficiency was found at 40% EtOH, with rates notably elevated in both the SDM (17.9%) and GRE (21.3%) regions. This outcome suggests that moderate ethanol concentration is optimal for extracting these compounds. Efficiency declined at elevated ethanol concentrations (80% and 100%), implying that certain extracted constituents may be related to non-polarity compounds. The P samples provided the highest extraction efficiencies across all organs and ethanol concentration conditions. The maximum extraction was observed with 40% EtOH in SDM (29.9%) and GRE (18.2%). These results indicate that crust extracts are abundant in both medium-polarity and non-polar components, which are most effectively solubilized at moderate EtOH concentrations. The SDM region retained high efficiency (29.6%) at 60% EtOH, while the GRE region demonstrated slightly increased efficiency at 22.6%. The substantial reduction in efficiency at 100% EtOH, particularly for GRE (6.1%), further validates the preference for aqueous ethanol systems in extracting bioactive substances. Overall, extraction efficiency from the seed samples was lower and presented more considerable variability. A distinct peak was found in the SDM extracts at 40% EtOH (28.0% efficiency), followed by a sharp drop to 80% (3.8%) and a return to baseline at 100% (11.1%). Extraction efficiency for the GRE samples remained consistently low, never surpassing 16.0%. Based on these results, specific components in organic seeds may be more sensitive to ethanol polarity or have unique solubility characteristics.

Liquid chromatography–tandem mass spectrometry (LC-MS/MS) has established itself as a powerful analytical technique and is extensively utilized across diverse disciplines, such as food science [21], medicine [22], pharmacy [23], environmental studies [24], and even research focused on human mental fatigue management [25]. This advanced method combines the separation capabilities of liquid chromatography with the precision and sensitivity of tandem mass spectrometry (MS/MS), allowing for the detection of low-abundance metabolites with greater confidence MS [26]. Therefore, LC-MS/MS-based metabolomics is an important approach in natural product research, supporting both the discovery of metabolites from complex mixtures or extracts and elucidating metabolic pathways. In this study, we utilized UHPLC-MS/MS to characterize the metabolites present in the fruits, pericarps, and seeds of *Z. piperitum* collected from both the SDM and GRE regions. Of the identified metabolites, the alkaloid group was most prevalent, comprising 29% and 41% in samples from SDM and GRE, respectively. Both organic acid and flavone contents were also found to be high in *Z. piperitum* from each geographic location. Previously, *Z. piperitum* was widely recognized as a rich source of alkaloids [20], as well as flavones and phenolic compounds [27]. Additionally, these predominant groups may contribute to the antioxidative properties of *Z. piperitum* extracts [28,29,30]. To discover the active constituents, we adopted a rapid screening approach that incorporated both liquid chromatography and the DPPH assay. This spectrophotometric assay offers a rapid and robust means for evaluating the overall antioxidant capacity of extracts, capitalizing on the strengths of liquid chromatography to effectively identify active components within plant extracts [13]. As a result, we identified seven active compounds from *Z. piperitum* collected from each region. The quantitative analysis of compounds identified from *Z. piperitum* extracts was calculated based on their peak areas compared to those of I.S. peak areas detected from chromatograms. Among identified compounds from the *Z. piperitum* extracts collected in the GRE region, compounds **2**, **13**–**15**, **17**–**22**, **36**, **37**, and **61** are major components with concentrations relative to I.S. ranging from 12.51 ± 0.01 to 5.09 ± 0.01 μg/mL (Table S5, Supplementary Materials). Among identified compounds from the *Z. piperitum* extracts collected in the SDM region, the identified compounds include **2**, **3**, **6**, **13**, **15**, **18**, **21**, **23**–**31**, **41**, **42**, and **49**, which are the major components, exhibiting concentrations relative to I.S. between 15.46 ± 0.05 and 5.21 ± 0.00 μg/mL (Table S6, Supplementary Materials). Among the active compounds identified from the GRE region, compounds **13**, **17**, **18**, and **61** were present in significant quantities with concentrations of 5.87 ± 0.06, 7.99 ± 0.02, 7.85 ± 0.01, and 8.54 ± 0.01 (μg/mL/I.S. concentration, Figure S12 and Table S5, Supplementary Materials), respectively, in the extract, indicating their crucial role in regulating the antioxidative effect. Similarly, the SDM extract demonstrated a significant presence of active compounds **13**, **15**, **23**, and **24** with concentrations of 9.60 ± 0.01, 9.03 ± 0.22, 15.46 ± 0.05, and 15.07 ± 0.03 (μg/mL/I.S. concentration, Figure S13 and Table S6, Supplementary Materials), respectively, highlighting their role in enhancing the antioxidative capacity of the extract. Furthermore, other chemical constituents were also identified from each extract.

Network pharmacology and molecular docking analyses identified critical targets linked to oxidative stress regulation, including EGFR, STAT3, AKT1, TNF, BCL2, CASP3, ESR1, PPARA, CYP19A1, and CDK2, which represented major nodes within the SDM- and GRE-PPI networks. These interactions suggest that the identified compounds may contribute to the antioxidant effects observed in vitro. These in silico analyses were performed to provide mechanistic support for the experimentally observed antioxidant effects, rather than to serve as independent confirmation. In network pharmacology, these genes coordinate cellular redox responses and cell fate during stress, homeostasis, inflammation, apoptosis, and survival. Among them, BCL2 protects mitochondria, reduces ROS accumulation, and prevents apoptosis [31]. ESR1 protects cells by enhancing mitochondrial dynamics and decreasing oxidative stress mediators via estrogen signaling [32]. PPARA induces antioxidant enzymes and regulates fatty acid oxidation, linking metabolism to oxidative resilience [33]. CYP19A1 indirectly affects the system by modulating estrogen biosynthesis, impacting antioxidant capacity [34]. CDK2 integrates oxidative signals into cell cycle regulation through oxidation [35]. By activating NADPH oxidases, mitochondrial dysfunction, and inflammatory pathways, EGFR signaling boosts ROS generation in oxidative diseases [36]. Downstream EGFR, STAT3 expression integrates oxidative and inflammatory signals via the JAK1/STAT3 axis, driving transcriptional programs associated with cytokine production, fibrosis, and metabolic adaptation. Under oxidative conditions, continuous activation of STAT3 encourages pro-inflammatory macrophage polarization and tissue fibrosis, as demonstrated in hepatic and renal models [37]. AKT1 mitigates oxidative-stress-induced injury by supporting antioxidant enzyme expression and maintaining mitochondrial integrity. However, its dysregulation may exacerbate ROS accumulation by negatively regulating Nrf2 pathways [38]. TNF links oxidative stress to inflammation via ROS-dependent TNFR1 activation, enhancing mitochondrial dysfunction and apoptosis, while also engaging protective TNFR2-mediated regenerative mechanisms [39]. Caspase-3 (CASP3) is a key executioner of apoptosis that is activated downstream of oxidative stress, mediating ROS-induced programmed cell death in damaged tissues. Dysregulated CASP3 activity contributes to the pathogenesis of oxidation-related diseases by promoting excessive tissue injury or, conversely, by enabling the survival of damaged cells when CASP3 activation is suppressed [40]. These genes intricately coordinate to maintain redox homeostasis, with disruptions leading to inflammation and disease, demonstrating their therapeutic relevance in targeting oxidation-related disorders. Therefore, understanding the individual and collective roles of these core targets offers promising avenues for developing effective antioxidative strategies.

Consequently, compound–target disease networks were constructed utilizing Cytoscape software, incorporating active components and antioxidant-related targets. These analyses revealed that the active components exhibited high degree scores due to extensive interactions with oxidant-related targets. Figure 3C and Figure 5C illustrate that each compound (node) may interact with multiple targets via various actions (edges), indicating complex and multifaceted relationships between these compounds and their targets. Network pharmacology analysis underscored the antioxidative potential of active metabolites in the SDM and GRE extracts, as reflected by their network topologies. Both metabolite–target interaction networks demonstrated that each compound was associated with several oxidation-related targets. This finding provides insight into how each active compound contributes to the antioxidative effects observed for the SDM and GRE extracts in the experimental assays. In the metabolite–target interaction network for SDM compounds, peak 15 (SDM) exhibited the most extensive interactions, linking to 56 oxidation-related targets (including key core targets: AKT1, EGFR, NFKB1, GSK3B) (Figure 2D). Among these, intersecting proteins for peaks 14 and 15 (SDM) were related to ROS generation and detoxification (NOO2, XDH), as well as responses to oxidative damage (AKR1B1). Additionally, peaks 10 and 15 both interacted with overlapping proteins, such as ESR1, ESR2, BACE1, CA4, and CA12, which play roles in oxidative regulation, amplification of oxidative stress, and modulation of ROS dynamics, respectively (Figure S3, Supplementary Materials). Peak 14 was found to interact with 34 proteins, including those involved in ROS generation (NOX4, ALOX5), redox-sensitive signaling (PRKCA, PRKCD, RPS6KA3), oxidative damage linked to inflammation (TNF, PTGS2, IL2, CD38, ELANE, ADORA1), as well as neurodegenerative and cardiovascular disease pathways (APP, MMP2, TERT, PDE5A, CA7). Peak 13 (SCM) established interactions with 36 proteins, which included those engaged in redox-sensitive regulation (STAT3, NOD2), oxidative inflammation (TLR9, IL2), and oxidative-stress-induced apoptosis (CASP3, CASP6, CASP7, CASP8, CASP9, BCL2L1). The GRE samples’ metabolite–target interaction network revealed that peak 27 interacted with 75 proteins associated with oxidative stress conditions (ESR1, ESR2, MTOR, CDK2, CDK4, PARP1, AGTR1, HMGCR, ABL1), redox-sensitive regulation (RAF1, BRAF, DYRK1A), oxidative inflammation (MMP13, MMP7, MMP14, PTGFR, SYK, SERPINE1), and diseases linked to oxidative processes (PSEN1, PSEN2, APH1A/B, NCSTN, DRD1, EPHA/EPHB, UPP1, DUT, KIF11). Similarly, peak 13 was associated with 47 proteins relevant to oxidative stress conditions (SLC1A2 (EAAT2), GRIK1/2/5, GRIA1/4, GRM1/2/4/5/7/8, FFAR1 (GPR40)), as well as proteins involved in oxidative inflammation (LTA4H, ADORA3, FABP3/4/5/2, SLC1A2 (EAAT2)) (Figure S4, Supplementary Materials). Therefore, these interaction networks illustrate the efficacy and potential therapeutic effects of each compound in treating oxidation-related diseases. Furthermore, *Z. piperitum* has demonstrated antioxidative properties in various in vitro models [41,42]. Peak 13 (GRE) exhibited a significant capacity to inhibit antioxidative enzymes, including SOD, TBARS, NO, and GSH-PX [43]. Peak 17 (GRE) was associated with beneficial activities on ABTS, FRAP, hyperlipidemia, gut microbiota, and obesity [44]. Peak 14 (GRE) demonstrated strong scavenging of DPPH radicals and contributed to reductions in ABTS and lipid peroxidation [45]. Peak 15 (SDM) elicited a moderate effect on DPPH radical scavenging activity [46]. Peak 23 (SDM) showed potent antioxidative and inhibitory potential relevant to Alzheimer’s disease [31]. Peak 24 (SDM) demonstrated antioxidative activity [47]. Peak 24 (SDM) demonstrated an antioxidative effect [47]. Moreover, this study established optimal extraction conditions considering both time-dependence and solvent use through ultrasonication, facilitating the development of standardized and fractionated extraction methods to yield products with high antioxidative capacity.

**Figure 9:**
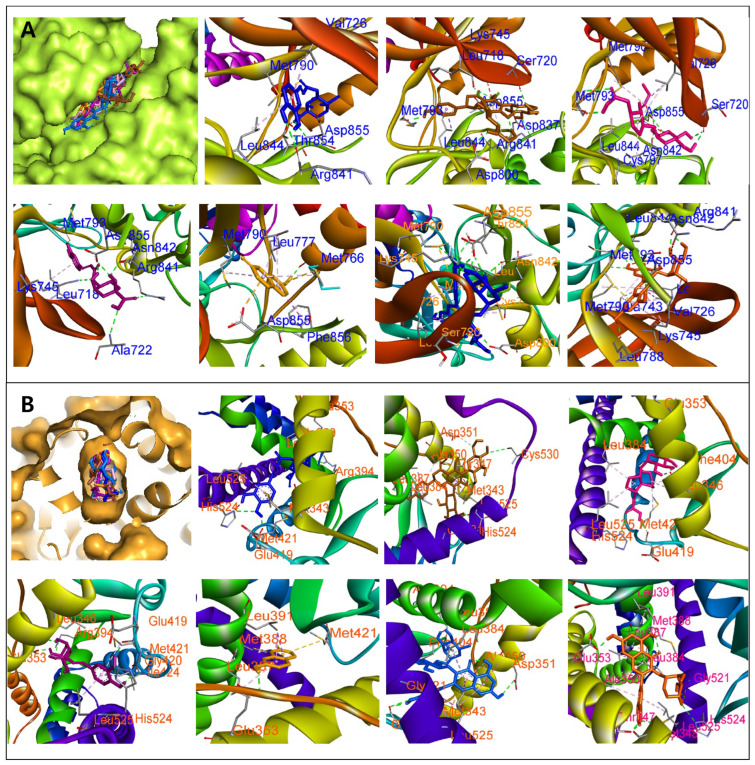
The 3D interactions of compounds 9 (blue), 10 (brown), 13 (pink), 14 (purple), 15 (dark yellow), 23 (marine), and 24 (orange) identified from *Z. piperitum* collected in the SDM region, with amino acids. They were docked into EGFR ((**A**): 5D41) and ESR1 ((**B**): 3ERT) proteins.

Molecular docking studies were conducted to verify the binding affinities of compounds identified from *Z. piperitum* extracts to key molecular targets. The results demonstrated that these compounds exhibit significant binding potential, as indicated by docking scores ranging from −13.10 to −4.27 kcal/mol. These interactions were further stabilized by the formation of hydrogen bonds and hydrophobic interactions with amino acids within the binding sites of the target proteins (Figure 9 and Figures S6–S10, Supplementary Materials). The molecular docking study also revealed that some compounds identified from the SDM location, including peaks 9, 14, 23, and 24, have substantial binding affinities for both ESR1 and EGFR, observed by their low docked scores. Among them, peak 9 exhibited several hydrogen-bond interactions with key residues of ESR1 protein, such as GLU353, LEU387, and ARG394, which are recognized as vital for ligand stability inside the receptor’s binding pocket [48]. Peaks 14, 23, and 24 also showed similar interaction patterns and formed hydrogen bonds with residues such as GLU419, HIS524, and GLY420. This suggests that they were occupied in the binding pocket of the ESR1 receptor. Furthermore, these compounds also interacted with the catalytic residue ASP855 of EGFR [49], which is a key residue of protein (Figure 9). Collectively, the active compounds exhibited antioxidant activity not only against radicals but also against reactive oxygen species produced by the LPS-activated RAW cells. The relative amounts of active compounds in the GRE region are higher than those found in SDM, proposing that the GRE extracts may possess a stronger antioxidative effect than the SDM extracts. These results indicate that active compounds in *Z. piperitum* have a solid crucial ability to core targets, suggesting their important roles in treating oxidation-related diseases.

## 4. Materials and Methods

### 4.1. Sample Preparation and Extraction

The fruits, pericarps, and seeds of *Z. piperitum*, cultivated by ‘Changssigojib’ company (Gurye, Jeonnam, Republic of Korea) were collected from different regions (SDM and GRE). All samples were authenticated for the species by Prof. Mina Lee, college of Pharmacy, Sunchon National University. They were stored as voucher specimens (SCNUP 39) at the Pharmacognosy Laboratory. These samples were dried in shade before being ground into powder, then sifted through a sieve (size 0.45 μm). Each sample was weighed at 1 g and added to 20 mL volume of EtOH in a ratio with water ranging from 0, 20, 40, 60, 80, to 100%, extracted by sonication (level 3.0) at room temperature (27 °C) for 120 min (repeated twice), respectively. Each extract solution was filtered through No. 2 Whatman filter paper (Whatman, Pleasanton, CA, USA) before being evaporated under a vacuum rotary at 40 °C. Finally, concentrated extracts were obtained and kept in the refrigerator at 4 °C.

Subsequently, the dried materials of the fruits, pericarps, and seeds were also weighed at 1 g and added to 20 mL of EtOH and water solvent systems including 0, 20, 40, 60, 80, and 100% EtOH in water, and extracted by sonication (level 3.0) during different times of 30, 60, 90, 120, 150 min at room temperature (27 °C), respectively, to investigate the extract efficiency depending on extraction time. The extract efficiency of each extract was calculated and presented in Table 3.

### 4.2. DPPH Radical Scavenging Assay

In the DPPH assay, each well contained 100 µL of sample volume, which was diluted in ethanol to final concentrations (10 and 100 µg/mL) and 100 µL of DPPH (200 µM) in an ethanol solution. The control was prepared with the same conditions as the amount of sample replaced by the addition of ethanol. All wells were mixed thoroughly and were incubated at room temperature for 30 min in the shade. When DPPH reacts with an antioxidant sample, the color conversion of deep violet into light yellow was measured at 517 nm with a micro reader (Epoch, Biotek Instruments, Winooski, VT, USA). Ascorbic acid (10, 100 µg/mL) (Sigma–Aldrich, Co., St. Louis, MO, USA) was used as a positive control. The percentage of DPPH reduction between the treated sample and negative control well was calculated with the following formula:

%EC = (A control − A sample) ∗ 100/(A control)

where A sample was the absorbance of the sample and A control was the absorbance of the untreated sample. Results are indicated as EC_50_, which corresponds to the sample concentration (µg/mL) required to inhibit by 50% of the initial DPPH radical scavenging activity under the given experimental conditions.

### 4.3. ABTS Radical Scavenging Assay

ABTS radical inhibitory activity was measured by mixing 100 µL of each sample solution (dissolved in distilled water) and 100 µL of ABTS solution (7 mM 2,20-azino-bis (3-ethylbenzothiazoline-6-sulfonic acid diammonium salt, ABTS, Sigma–Aldrich, Co., St. Louis, MO, USA) mixed with 2.45 mM potassium persulfate). After incubating at room temperature for 20 min, the absorbance of the mixture was measured at 734 nm. Ascorbic acid (10, 100 µg/mL) was used as the positive control:

%EC = (A control − A sample) ∗ 100/(A control)

where A sample was the absorbance of the sample and A control was the absorbance of the untreated sample. Similarly, the results are indicated as EC_50_, which corresponds to the sample concentration (µg/mL) required to inhibit by 50% of the initial ABTS radical scavenging activity under the given experimental conditions.

### 4.4. ROS Assay

#### 4.4.1. Cell Culture and Viability

RAW264.7 cells were obtained from the Korean Cell Line Bank in Seoul, Republic of Korea. Cells were kept at 37 °C in Dulbecco’s Modified Eagle’s Medium (DMEM) with 10% heat-inactivated fetal bovine serum (FBS), 100 µg/mL streptomycin sulfate, and 100 IU/mL penicillin in a humidified environment with 5% CO_2_. RAW264.7 cells were planted in 6-well plates at a density of 1 × 10^4^ cells/well and incubated for 20 h. Cells were treated with either a positive control (ascorbic acid) or 0%, 20%, 40%, 60%, 80%, and 100% extracts of fruits and pericarps at concentrations ranging from 10 to 100 µg/mL. After 2 h of pretreatment, 100 ng/mL of lipopolysaccharide (LPS) was given to the cells, and they were cultured for 20 h [12]. After incubation, 5 mg/mL of MTT solution was added to each well in the plates, and the plates were incubated for an extra 4 h at 37 °C to enable the production of formazan crystals. To solubilize the formazan, 100 µL of dimethyl sulfoxide (DMSO; Sigma-Aldrich, St. Louis, MO, USA) was added to each well after the supernatants were carefully removed. The plates were incubated at room temperature for fifteen minutes after being gently shaken. The absorbance was then measured with a microplate reader (BioTek Instruments, Winooski, VT, USA) at 570 nm.

#### 4.4.2. ROS Determinations

To assess ROS production under the same treatment conditions, cells were carefully washed with PBS and incubated with 10 µM DCFH-DA in serum-free medium (diluted 1:1000) for 20 min at 37 °C in the dark. Fluorescence intensity, corresponding to ROS levels, was measured at an excitation/emission wavelength of 485 and 530 nm using a microplate reader.

### 4.5. Analytical Method and Metabolomic Procedures

All samples of *Z. piperitum* were analyzed by UHPLC-Orbitrap-MS/MS (Thermo Fisher Scientific, Gangnam-gu, Seoul, Republic of Korea) referring to our previous methods [50]. The stational phase was Waters Acquity UPLC HSS T3 column (4.6 × 100 mm, 1.8 μm, Waters, Milford, MA, USA) maintained at 40 °C. Flow rate was set at 0.2 mL/min and each 2 μL of sample was injected into the UHPLC system. Mobile phases consisted of channel A (high grade water buffered with 0.01% ammonia) and channel B (acetonitrile buffered with 0.01% ammonia). Eluent conditions were 0 min (3% B)–3 min (3% B)–4 min (16% B)–9 min (16% B)–12 min (55% B)–21 min (63% B)–23 min (100% B)–25 min (100% B)–26 min (3% B)–30 min (3% B). The column was re-equivalent to initial condition for 5 min before injection of next sample. Mass spectra were obtained by the settings as follows: Samples were ionized by electrospray ionization. Mass spectra were collected in both negative and positive ion modes. Mass scanning was set in range 100–1500 *m*/*z*, with spay voltage for positive (3500 V) and negative (2500 V). The sheath gas flow rate was 50 arbitrary units, with an auxiliary gas flow rate of 15 arbitrary units. The heating temperature was maintained at 325 °C and the vaporizer temperature was 350 °C. HCD collision energies (%) were set at 15, 30, and 60; Orbitrap resolution was 120,000. Additionally, an internal standard (caffeic acid, I.S.) was spiked with a known amount into the extract solution, following the above analytical procedure, to determine the relative quantities of peaks detected from the extract chromatograms. The relative peak area between each compound and the I.S. peaks was determined by using the following equation:

Relative peak area (%) = (Compound peak area ∗ 100)/I.S. peak area

The relative quantitation of each compound was calculated by using the following equation:

Compound amount (μg/mL) = Relative peak area ∗ 15/100

where the I.S. concentration was 15 μg/mL.

Quantitation of compounds detected from chromatographic profiles of *Z. piperitum* was classified into major, minor, or trace components based on their amount as shown in Tables S5 and S6 (Supplementary Materials).

### 4.6. LC-DPPH Screening Antioxidant from SDM and GRE Extracts

Each extract was prepared at a concentration of 4.0 mg/mL. An amount of 100 μL of the sample was mixed with 100 μL of the DPPH solution (0.2 mg/mL). The control sample was prepared by replacing DPPH with the same volume of MeOH. The reaction mixture was incubated for 30 min at 37 °C by using a water bath in the shade [13]. After the reaction was completed, the control and reaction mixture were analyzed by using the above analytical methods to assess the antioxidants of the *Z. piperitum* samples.

### 4.7. Data Processing and Compound Annotation

Raw file data were imputed into Mzmine version 3.9.0 [51] before uploading to the GNPS platform to generate the GNPS-FBMN data file. Additionally, the compounds were also identified by analysis using MS-DIAL (version 5.1.230912) software in cooperation with the mass database (Mass American, MoNa mass library). The GNPS-FBMN was visualized by using Cytoscape (ver 3.10) software [50].

### 4.8. Network Pharmacology

#### 4.8.1. Prediction of Compounds and Oxidation-Related Targets

To investigate the potential effects of active compounds obtained from LC-DPPH, these compounds were input into the Pubchem (https://pubchem.ncbi.nlm.nih.gov/, accessed on 28 May 2025). Then, they were subjected to the Swisstargetprediction (https://www.swisstargetprediction.ch/, accessed on 2 June 2025) by using their Smiles, selecting *Homo sapiens* as the species, followed by other default settings of the webserver [52]. The oxidation-related targets were retrieved from the Gene Cards web-platform (https://www.genecards.org, accessed on 10 June 2025) and DisGeNET web-platforms [53] by entering the keyword “Oxidation”. To identify the intersection of compound targets and oxidation-related targets, Venny (version 2.1.0, https://bioinfogp.cnb.csic.es/tools/venny/, accessed on 12 June 2025) was used to ascertain the genes.

#### 4.8.2. PPI Network Construction

The intersecting targets were imported into the STRING database (https://string-db.org/) at a confidential score above 0.7 before exporting the output data to the Cytoscape (version 3.10.1) software for analyzing and visualizing the target interconnection. The topological features of the PPI network were analyzed, and hub genes were identified based on the intersection of their central scores using the Cytohubba plug-in in the Cytoscape program.

#### 4.8.3. Construction of Compound–Target Disease Network

The shared targets obtained from the above Venn online platform were interrupted with key oxidants from *Z. piperitum* extracts to create the interrelationships between compound, targets, and oxidation pathways by using the above Cytoscape visualization of the interactions and key compounds based on their degree score distribution in the interaction network [54]. The high score reflected the importance of node in the compound–target disease network.

### 4.9. Molecular Docking

The RCSB protein data bank (https://www.rcsb.org; accessed on 24 October 2025) provided the protein’s 3D structures [PDB ID: BCL2 (6O0K), CDK2 (4FX3), CYP19A1 (3EQM), ESR1 (3ERT), PPARA (2ZNN), AKT1 (1H10), CASP3 (2XYG), EGFR (5D41), STAT3 (6NJS), and TNF (2AZ5)]. MGL tools 1.5.6 were used to produce the proteins and ligand. To process the receptor structures, polar hydrogen atoms, Kollman charges, and water were added. SDF files of the structures of a few chosen compounds were obtained from PubChem (https://pubchem.ncbi.nlm.nih.gov). The Open Babel was then used to convert the geometry of these structures into pdbqt format. Gasteiger charges were added to the ligand to change its conformation. Pymol was utilized to determine a grid box of coordinates. The Lamarckian genetic algorithm (4.2) was employed to explore conformations, configured with 10 docking runs, a maximum number of generations of 27,000, a population size of 150 dockings, a maximum energy evaluation of 25 million, and random initial positions and conformations. The dimensions of the grid box were centered on the cognate ligand, serving as a spacing grid that encompassed the entire binding site, and were established as follows: 6O0K, centers −14.226 Å × 1.146 Å × −10.800 Å along with dimensions 46 Å × 56 Å × 56 Å and spacing of 0.375 Å; 2ZMN, centers 10.565 Å × 3.288 Å × −8.990 Å along with dimensions 40 Å × 40 Å × 40 Å and spacing of 0.375 Å; 1H10, centers 17.070 Å × 20.245 Å × 13.360 Å along with dimensions 40 Å × 48 Å × 62 Å and spacing of 0.375 Å; 2XYG, centers 36.356 Å × 38.924 Å × 32.077 Å along with dimensions 40 Å × 40 Å × 34 Å and spacing of 0.375 Å; 5D41, centers −31.801 Å × 33.574 Å × 14.596 Å along with dimensions 62 Å × 44 Å × 60 Å and spacing of 0.375 Å; 3EQM, centers −83.350 Å × 49.601 Å × 50.600 Å along with dimensions 50 Å × 64 Å × 78 Å and spacing of 0.375 Å; 3ERT, centers 31.910 Å × −1.800 Å × 25.170 Å along with dimensions 40 Å × 40 Å × 40 Å and spacing of 0.375 Å; 6NJS, centers 13.498 Å × 54.117 Å × 0.100 Å along with dimensions 72 Å × 74 Å × 48 Å and spacing of 0.375 Å; 2AZ5, centers −19.573 Å × 74.852 Å × 33.838 Å along with dimensions 50 Å × 50 Å × 50 Å and spacing of 0.375 Å; 4FX3, centers −19.573 Å × 74.852 Å × 33.838 Å along with dimensions 50 Å × 50 Å × 50 Å and spacing of 0.375 Å. AutoDock 4.2.6 was used for the protein–ligand docking computations. The docking protocol was validated by redocking the native ligand exhibiting the RMSD less than 2 Å. The RMSD values were determined as 1.19 Å (2ZNN), 1.31 Å (2X08), 0.58 Å (3EQM), 1.53 Å (3ERT), 0.82 Å (4FX3), 0.88 V (2AZ5), 1.83 Å (2XYG), 1.54 Å (5D41), and 1.18 Å (6NJS). Moreover, active compounds identified from the SDM extracts were also docked into the binding sites of the PH domain of the AKT1 protein [55]. Then, an analysis of all selected compounds was performed to calculate binding energies. Discovery Studio 2021 was used to visualize the interactions between residues and ligands.

### 4.10. Statistical Analysis

Data collected from at least three different experiments were expressed as the mean ± standard error of the mean (n = 3). Dunnett’s post hoc test and a one-way analysis of variance (ANOVA) were used to examine statistically significant data using GraphPad Prism (version 8.0) software (GraphPad Software, Inc., San Diego, CA, USA). Differences were significant at * *p* < 0.05 and ** *p* < 0.001 when compared to controls.

## 5. Conclusions

All extractable compounds present in different organs of *Z. piperitum* cultivated in the Sangdong-myeon and Gurye-eup regions of Korea were identified via UHPLC-MS/MS virtual analysis using a mass database. *Z. piperitum* pericarps exhibited potent antioxidant properties, demonstrated by the highest radical scavenging activities, and oxidative components were validated through compound–oxidation target network and molecular docking analyses. These results highlight the potential of *Z. piperitum* as a natural antioxidative agent for health promotion. Additional research should be performed to confirm the antioxidative effects using animal models, and further investigation into the bioactive compounds present in *Z. piperitum* may uncover other pharmacological advantages. Our findings suggest that the pericarps of this plant may be utilized to develop manufacturing processes for functional products aimed at improving human health and for therapeutic approaches to oxidative disorders.

## Figures and Tables

**Table 1:** Antioxidative effect of samples via DPPH and ABTS radical scavenging activity.

**Organ**	**Location**	**SDM**	**GRE**
	**Extract (EtOH, %)**	**ABTS (EC_50_, μg/mL)**	
F	0	117.2	147.4
	20	62.1	104.3
	40	60.9	81.8
	60	61.5	84.9
	80	89.8	91.8
	100	73.3	480.5
P	0	75.1	77.4
	20	72.9	89.2
	40	75.4	76.8
	60	55.9	78.5
	80	58.6	114.4
	100	71.3	160.9
S	0	301.5	315.9
	20	519.7	441.2
	40	643.8	300.7
	60	638.8	715.9
	80	210.5	193.2
	100	967.2	893.1
A.A.	Pos.	7.5	7.9
**Organ**	**Location**	**SDM**	**GRE**
	**Extract (EtOH, %)**	**DPPH (EC_50_, μg/mL)**	
F	0	184.1	352.4
	20	98.5	137.3
	40	98.1	81.6
	60	91.8	104.4
	80	85.5	100.4
	100	118.1	621.4
P	0	80.1	64.5
	20	60.5	93.2
	40	78.3	64.1
	60	48.0	62.5
	80	67.4	141.3
	100	71.1	158.5
S	0	666.3	483.6
	20	242.1	236.8
	40	840.1	1012.6
	60	900.3	1084.5
	80	421.2	347.7
	100	1011.7	783.3
A.A.	Pos.	5.9	5.9

**Table 2:** Antioxidative effect of samples against ROS production secreted on RAW264.7 cells.

Organ	Location	SDM	GRE
	Extract (EtOH, %)	(IC_50_, μg/mL)	
F	0	729.4	471.9
	20	100.0	97.1
	40	87.2	43.7
	60	87.9	51.2
	80	70.9	58.5
	100	38.0	34.4
P	0	102.3	80.1
	20	92.6	32.4
	40	84.7	30.1
	60	49.9	12.0
	80	57.1	10.1
	100	17.9	9.1
A.A.	Pos.	10.6	10.6

**Table 3:** The extraction efficiency of the pericarps, fruits, and seeds collected from the SDM and GRE regions.

Organ	Extract (EtOH, %)	Yield (mg)	SDM (%)	Yield (mg)	GRE (%)
F	0	123.0	12.3	111.8	11.2
	20	149.0	14.9	145.0	14.5
	40	179.1	17.9	212.9	21.3
	60	139.2	13.9	157.1	15.7
	80	135.3	13.5	132.8	13.3
	100	-	-	-	-
P	0	188.9	18.9	151.9	15.2
	20	270.8	27.1	191.2	19.1
	40	299.0	29.9	182.0	18.2
	60	295.9	29.6	225.8	22.6
	80	228.1	22.8	175.0	17.5
	100	124.2	12.4	61.2	6.1
S	0	110.8	11.1	119.7	12.0
	20	152.0	15.2	102.3	10.2
	40	279.8	28.0	159.6	16.0
	60	205.2	20.5	135.0	13.5
	80	37.7	3.8	45.1	4.5
	100	110.8	11.1	37.2	3.7

## Data Availability

Data are contained within the article and Supplementary Material.

## Supplementary Materials

The following supporting information can be downloaded at: https://www.mdpi.com/article/10.3390/plants15010148/s1, Figure S1: Cytotoxic effect of samples collected in SDM (A) and GRE (B) locations on RAW264.7 cells; Figure S2: Chemical profiles of SDM_P extracts treated and non-treated with DPPH; Figure S3: Chemical profiles of GRE_P extracts treated and non-treated with DPPH; Figure S4: Metabolite–target interaction network of active compounds identified from SDM samples; Figure S5: Metabolite–target interaction network of active compounds identified from GRE samples; Figure S6: 3D Interactions of compounds 13 (marine), 17 (orange), 18 (forest), 27 (magenta), 39 (blue), 46 (violet), 61 (lemon), identified from *Z. piperitum* collected in the GRE region, with amino acids. These compounds were docked into PPARA (A: 2ZNN) and CYP19A1 (B: 3EQM) proteins; Figure S7: 3D Interactions of compounds 13 (marine), 17 (orange), 18 (forest), 27 (magenta), 39 (blue), 46 (violet), 61 (lemon), identified from *Z. piperitum* collected in the GRE region, with amino acids. These compounds were docked into ESR1 (3ERT) protein; Figure S8: 3D Interactions of compounds, 9 (blue), 10 (brown), 13 (pink), 14 (purple), 15 (dark yellow), 23 (marine), 24 (orange) identified from *Z. piperitum* collected in the SDM region, with amino acids. They were docked into TNF-α (A: 2AZ5) and CASP3 (B: 2XYG) proteins; Figure S9: 3D Interactions of compounds 13 (marine), 17 (orange), 18 (forest), 27 (magenta), 39 (blue), 46 (violet), 61 (lemon), identified from *Z. piperitum* collected in the GRE region, with amino acids. These compounds were docked into BCL2 (A: 6O0K) and CDK2 (B: 4FX3) proteins; Figure S10: 3D Interactions of compounds, 9 (blue), 10 (brown), 13 (pink), 14 (purple), 15 (dark yellow), 23 (marine), 24 (orange) identified from *Z. piperitum* collected in the SDM region, with amino acids. They were docked into STAT3 (A: 6NJS) and AKT1 (B: 1H10) proteins; Figure S11: Chemical profiles of internal standard (A, caffeic acid: I.S.), and SDM (B) and GRE extracts spiked with internal standard (15 μg/mL); Figure S12: Relative quantities of compounds in relation to the internal standard detected from chromatogram extracts of *Z. piperitum* collected in the GRE region; Figure S13: Relative quantities of compounds in relation to the internal standard detected from chromatogram extracts of *Z. piperitum* collected in the SDM region; Table S1: Extraction efficiency depends on extraction time; Table S2: Peak reduction of active compounds from LC-DPPH; Table S3: Identified compounds detected from the GRE region; Table S4: Identified compounds detected from the GRE region; Table S5: Quantitation of identified compounds from *Z. piperitum* collected in the GRE region; Table S6: Quantitation of identified compounds from *Z. piperitum* collected in the SDM region.
