# Supplementary material for: Integrating Molecular Analysis and the Pharmacology Network to Discover the Antioxidative Effects of Zanthoxylum piperitum Fruits
Source: Plants (Basel). 2026 Jan 4;15(1):148. doi: 10.3390/plants15010148 (PMC12787408; doi:10.3390/plants15010148)
Supplement: Supplementary file 1 [file plants-15-00148-s001.zip › plants-3975968-supplementary.pdf]

Supplementary materials

Integrating molecular and pharmacology network to discover anti-oxidative effect of *Zanthoxylum piperitum* fruits

Ducdat Le [1,2], Thinhulinh Dang[1], Thientam Dinh[1], Sujeong Yu[1], Vinhquang Truong[2] and Mina Lee [1,2,]*

[1] College of Pharmacy and Research Institute of Life and Pharmaceutical Sciences, Sunchon National University, Suncheon-si 57922, Republic of Korea; ddle@scnu.ac.kr (D.D.L.); quangvtruong00@gmail.com (V.T.); minalee@scnu.ac.kr (M.L.)

[2] Department of Natural Cosmetics Science and Smart Beautytech Research Institute, Sunchon National University, Suncheon-si 57922, Republic of Korea; ddle@scnu.ac.kr (D.D.L.); quangvtruong00@gmail.com (V.T.); minalee@scnu.ac.kr (M.L.)

*Corresponding author:
Mina Lee, minalee@scnu.ac.kr; Tel.: +82-61-750-3764; Fax: +82-61-750-3708

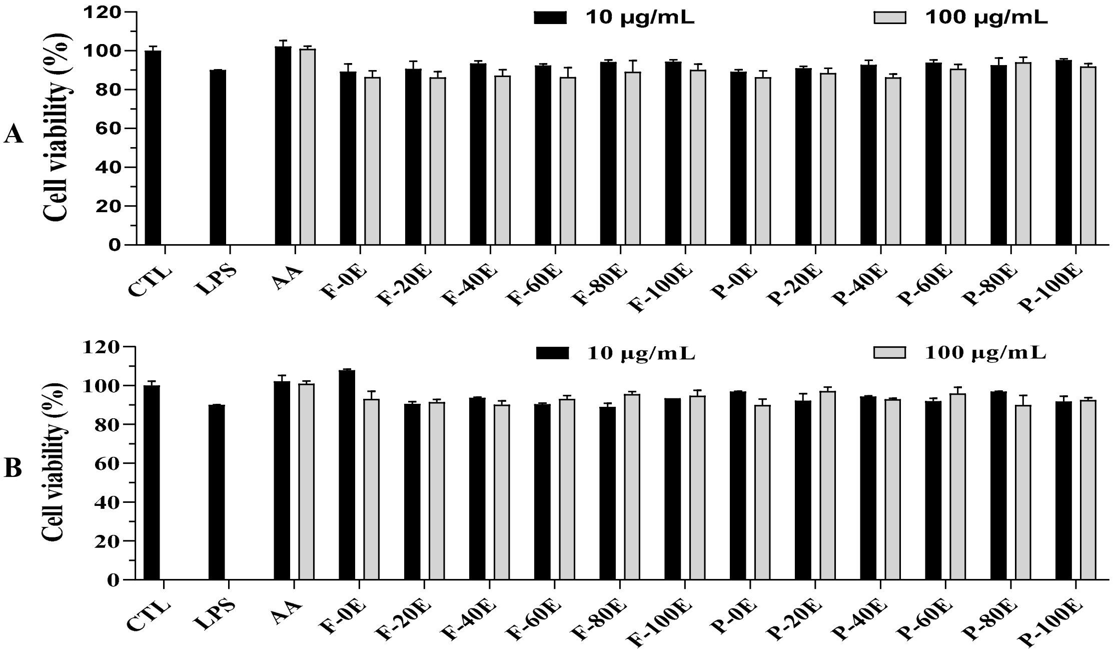

Figure S1. Cytotoxic effect of samples collected in SDM (A) and GRE (B) regions on RAW264.7 cells. Samples include fruits (F), pericarps (P), and seeds (S), which were extracted by using mixture solvent of ethanol (E) ratio (0%, 20%, 40%, 60%, 80%, and 100%) in water.

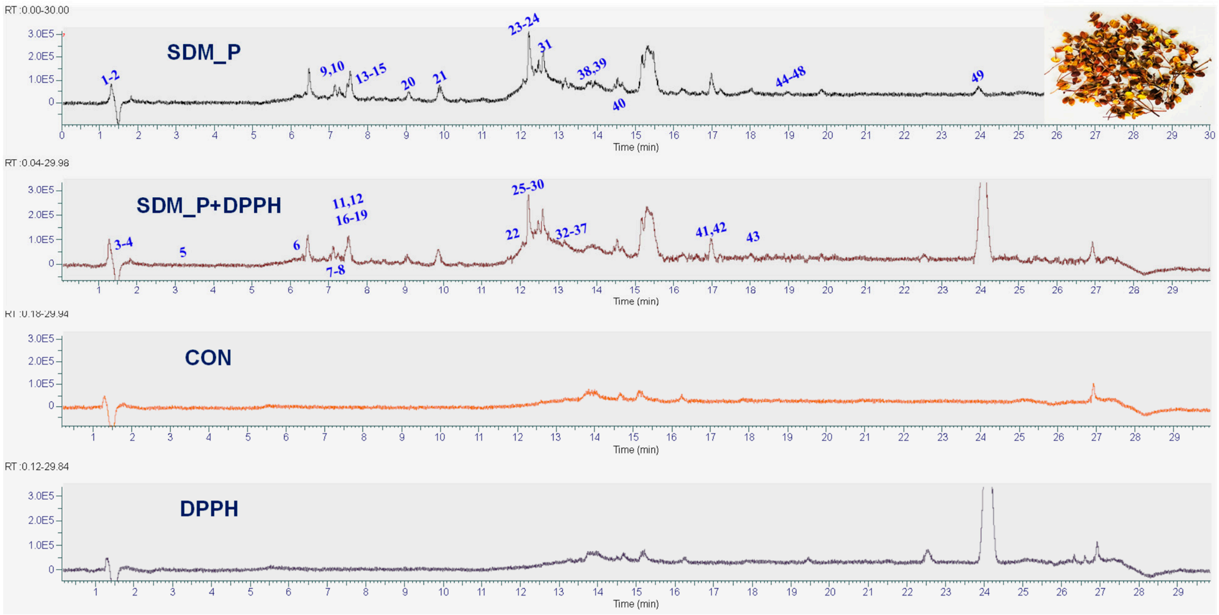

Figure S2. Chemical profiles of SDM_P extracts treated and non-treated with DPPH.

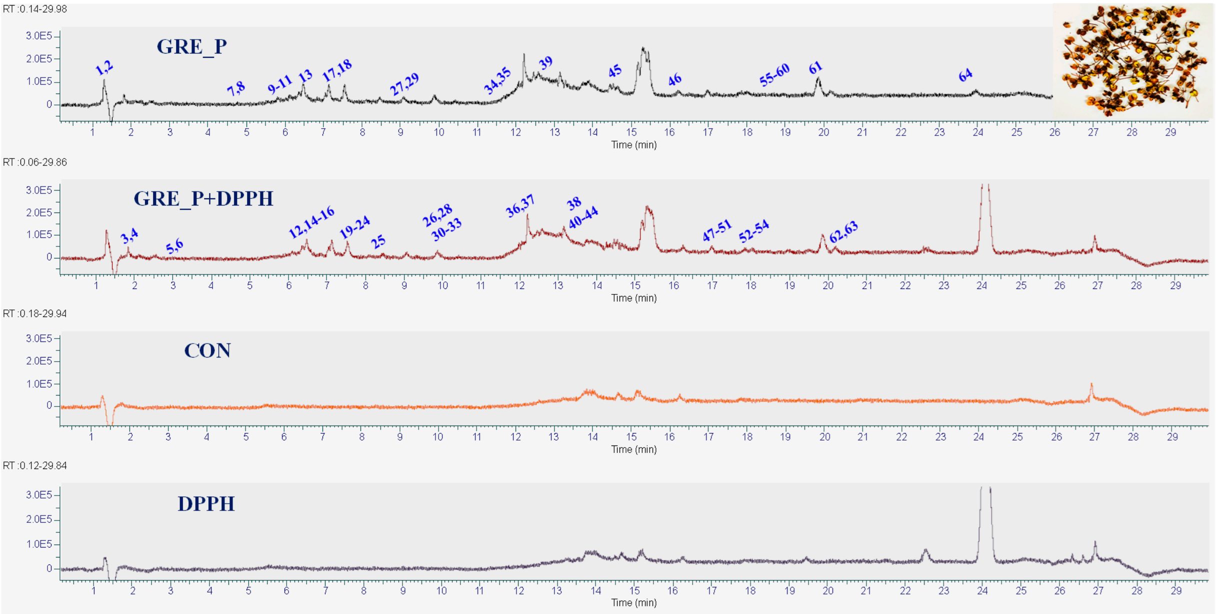

Figure S3. Chemical profiles of GRE_P extracts treated and non-treated with DPPH.

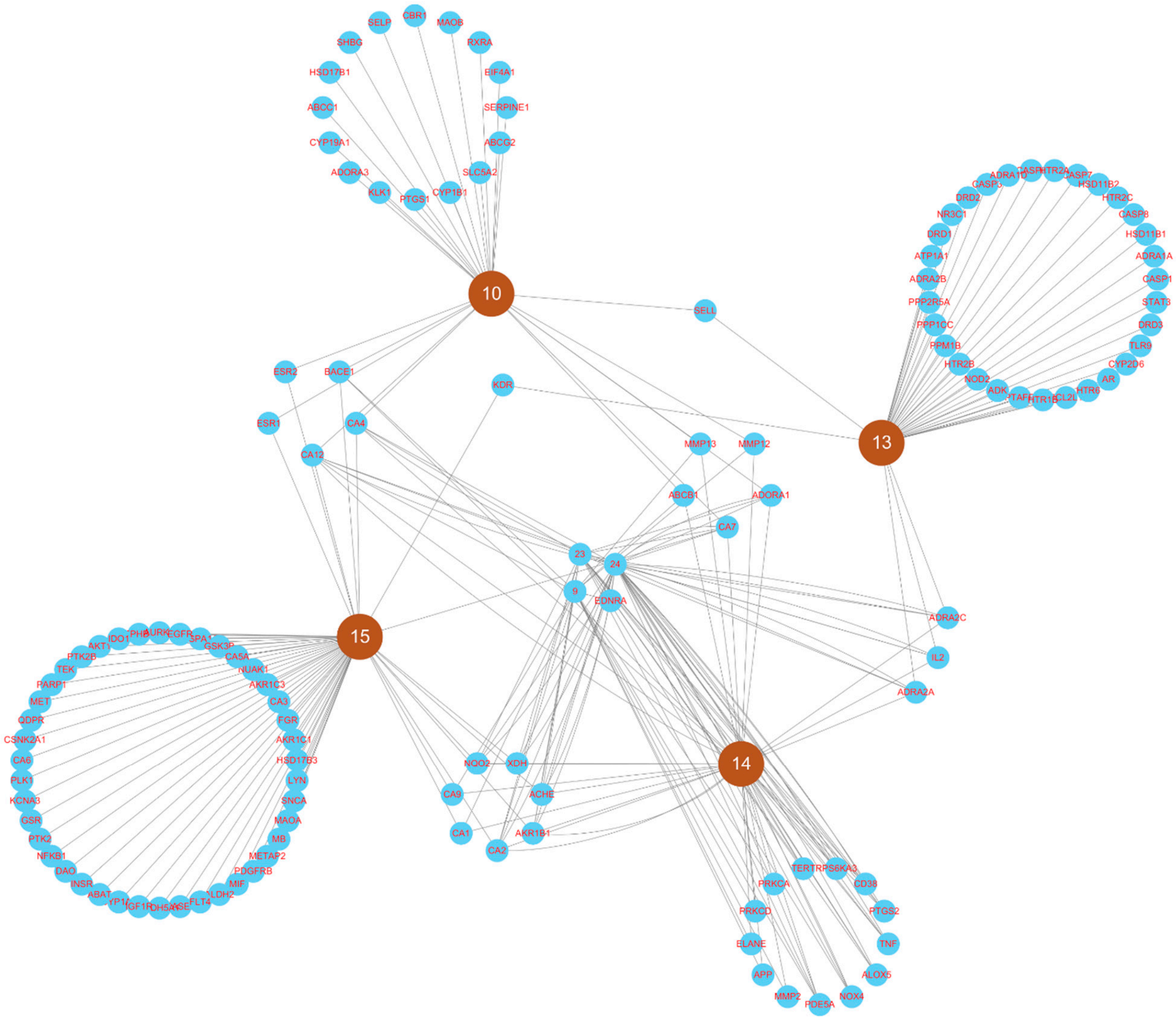

Figure S4. Metabolite-target interaction network of active compounds identified from SDM sample.

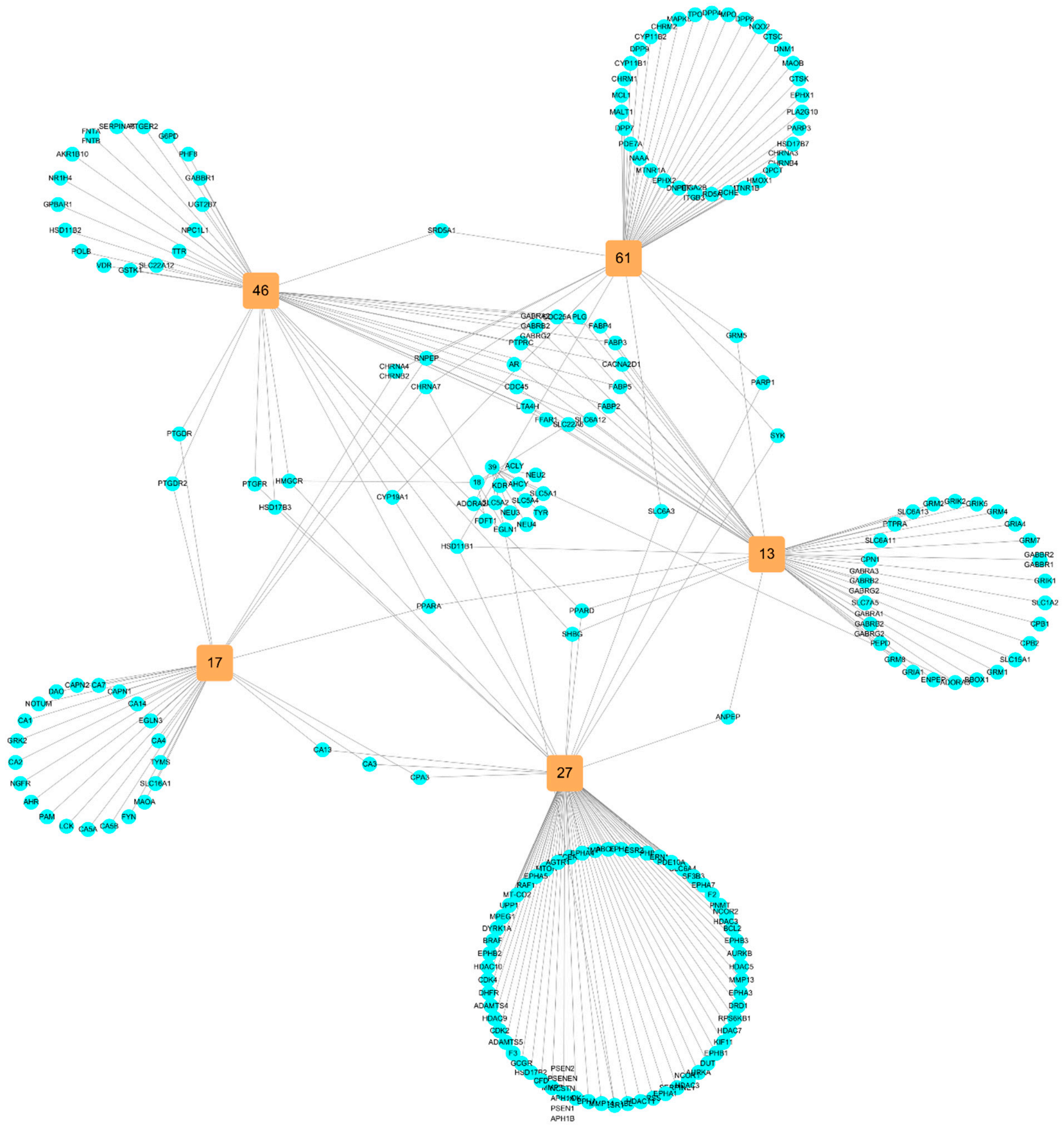

Figure S5. Metabolite-target interaction network of active compounds identified from GRE sample.

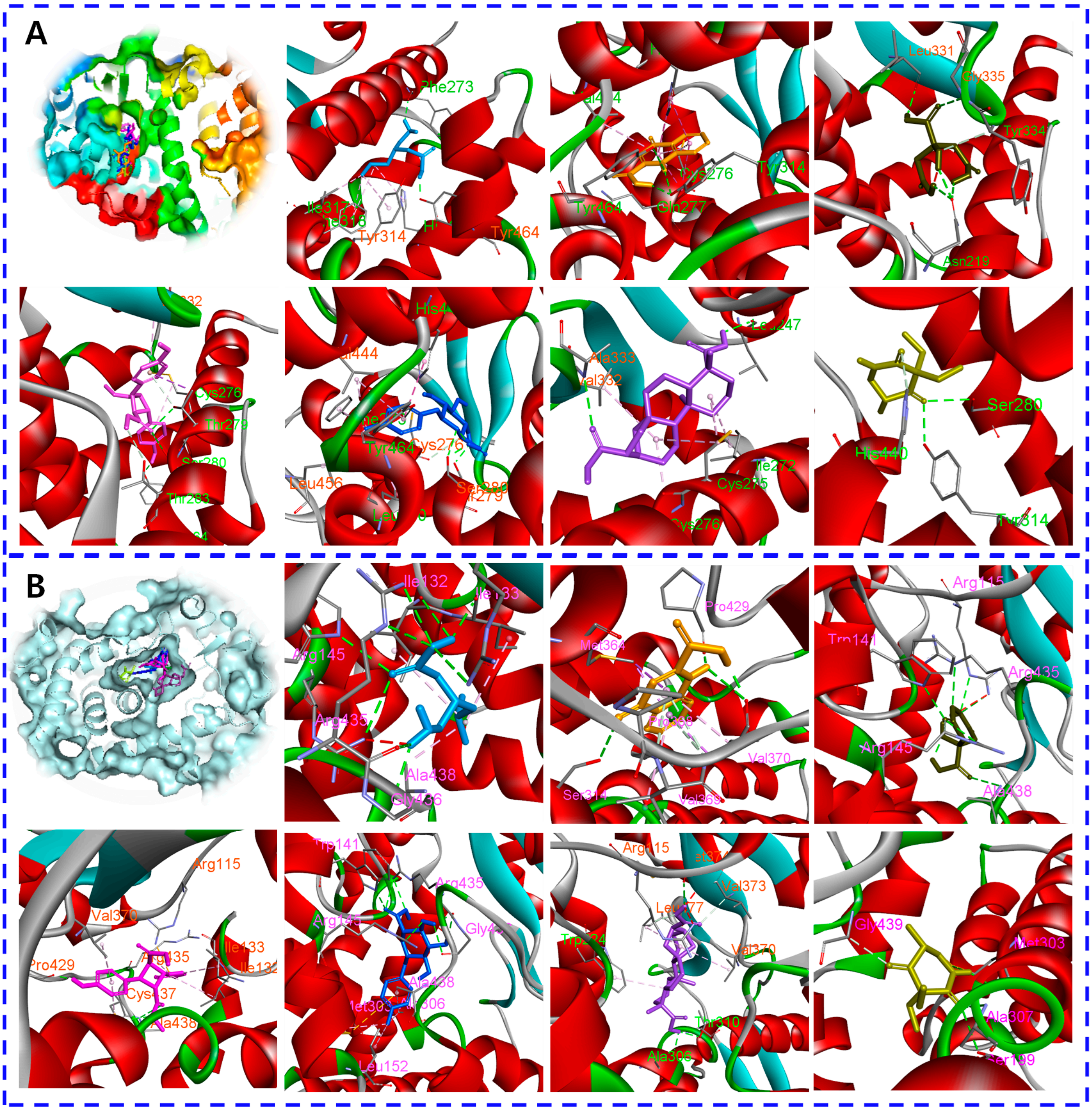

Figure S6. 3D Interactions of compounds 13 (marine), 17 (orange), 18 (forest), 27 (magentas), 39 (blue), 46 (violet), 61 (limon), identified from Z. piperitum collected in GRE region, with amino acids. These compounds were docked into PPARA (A: 2ZNN) and CYP19A1 (B: 3EQM) proteins.

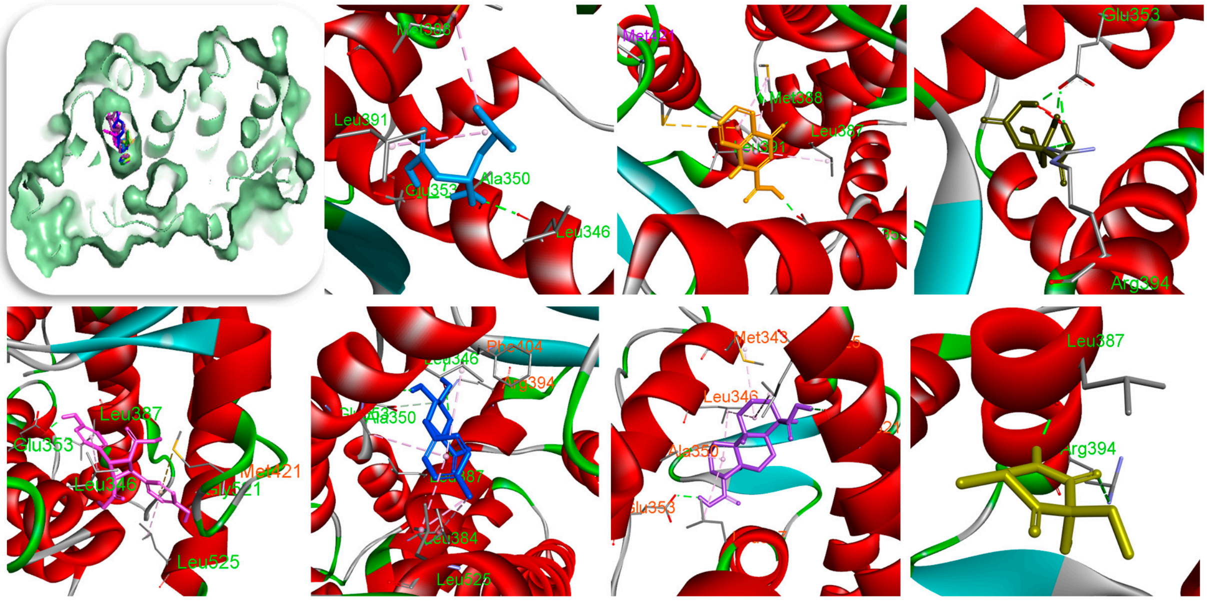

Figure S7. 3D Interactions of compounds 13 (marine), 17 (orange), 18 (forest), 27 (magentas), 39 (blue), 46 (violet), 61 (limon), identified from *Z. piperitum* collected in GRE region, with amino acids. These compounds were docked into ESR1 (3ERT) protein.

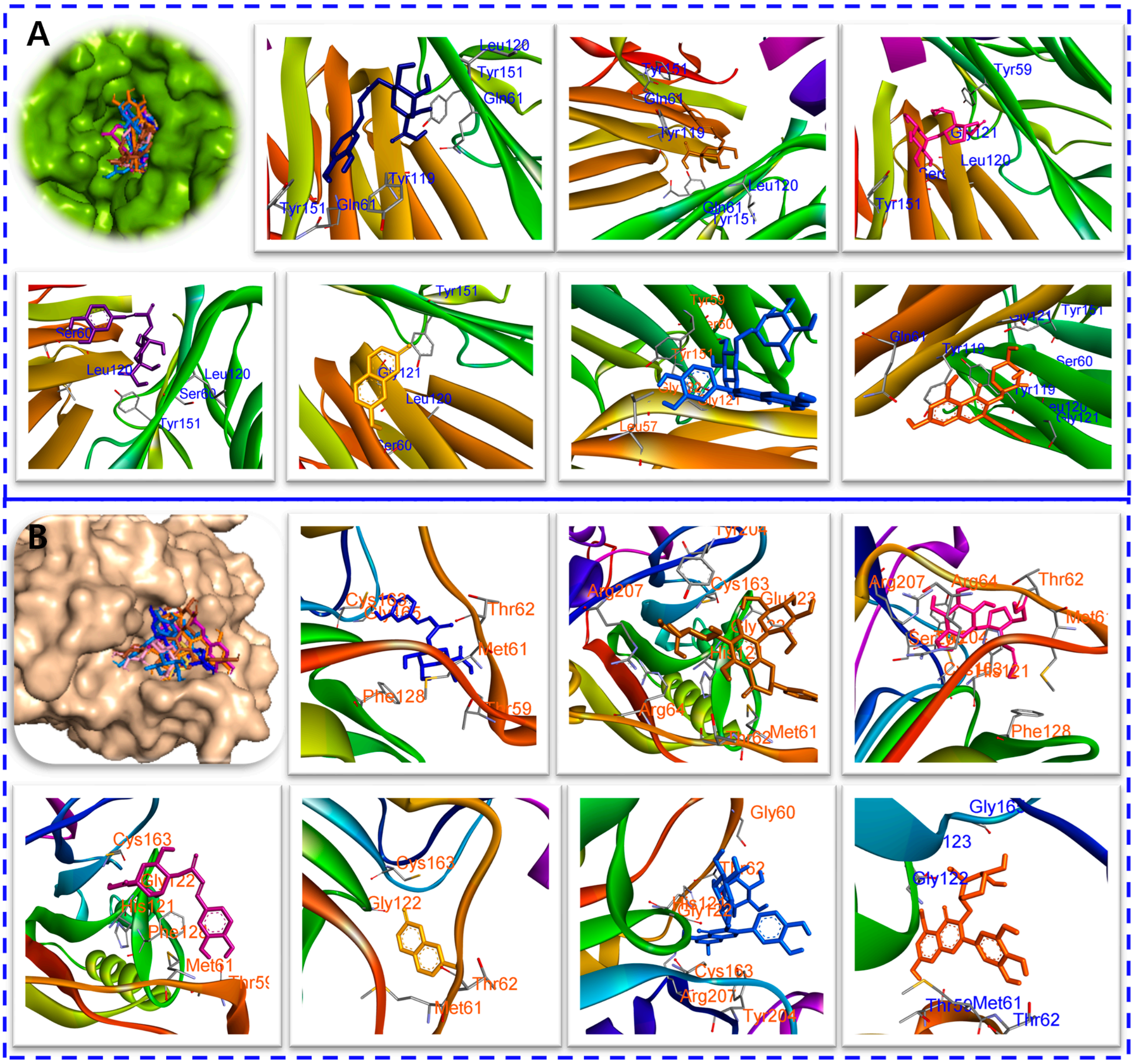

Figure S8. 3D Interactions of compounds, **9** (blue), **10** (brown), **13** (pink), **14** (purple), **15** (dark yellow), **23** (marine), **24** (orange) identified from *Z. piperitum* collected in SDM region, with amino acids. They were docked into TNF-α (A: 2AZ5) and CASP3 (B: 2XYG) proteins.

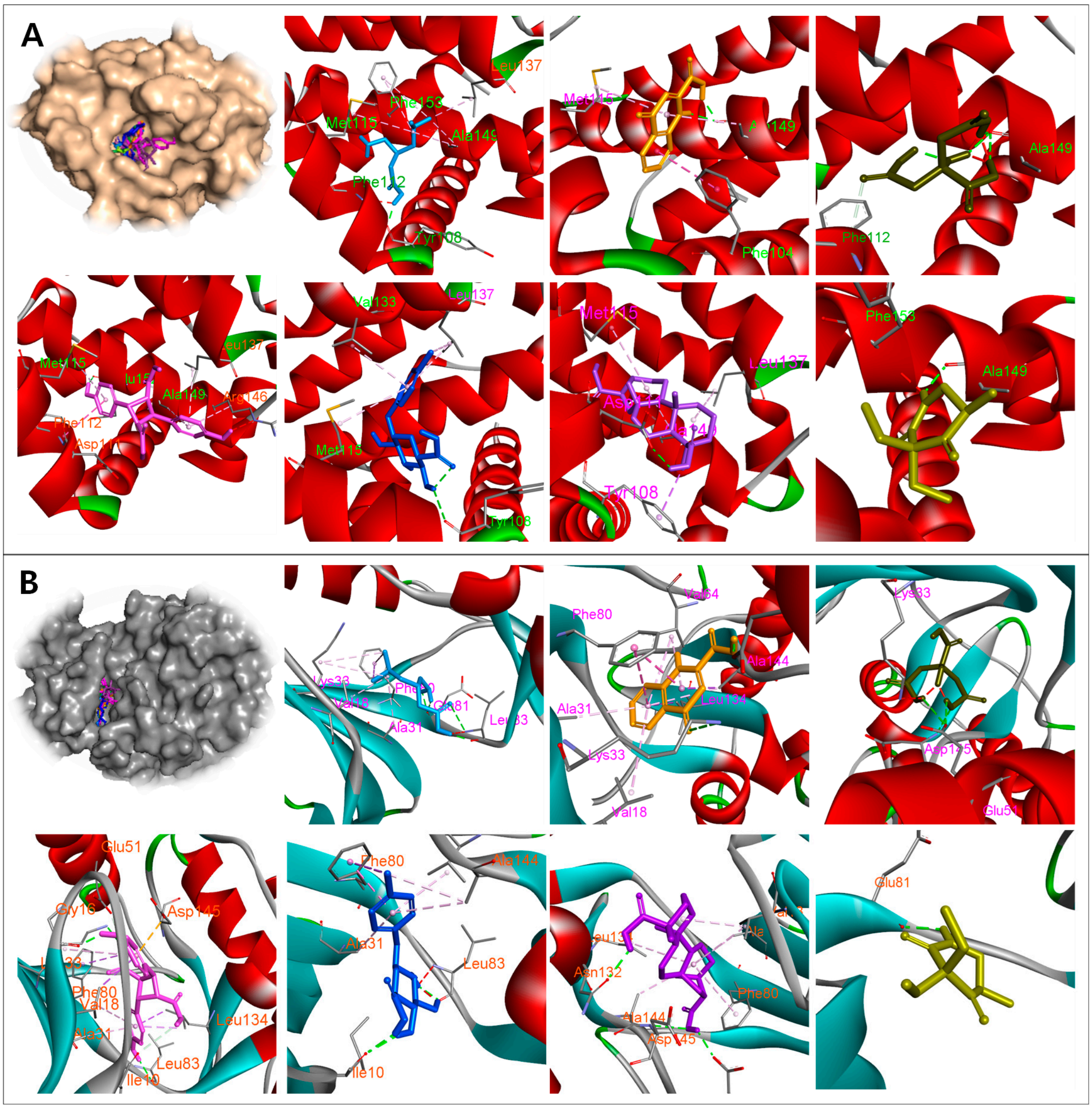

Figure S9. 3D Interactions of compounds 13 (marine), 17 (orange), 18 (forest), 27 (magentas), 39 (blue), 46 (violet), 61 (limon), identified from *Z. piperitum* collected in GRE region, with amino acids. These compounds were docked into BCL2 (A: 6O0K) and CDK2 (B: 4FX3) proteins.

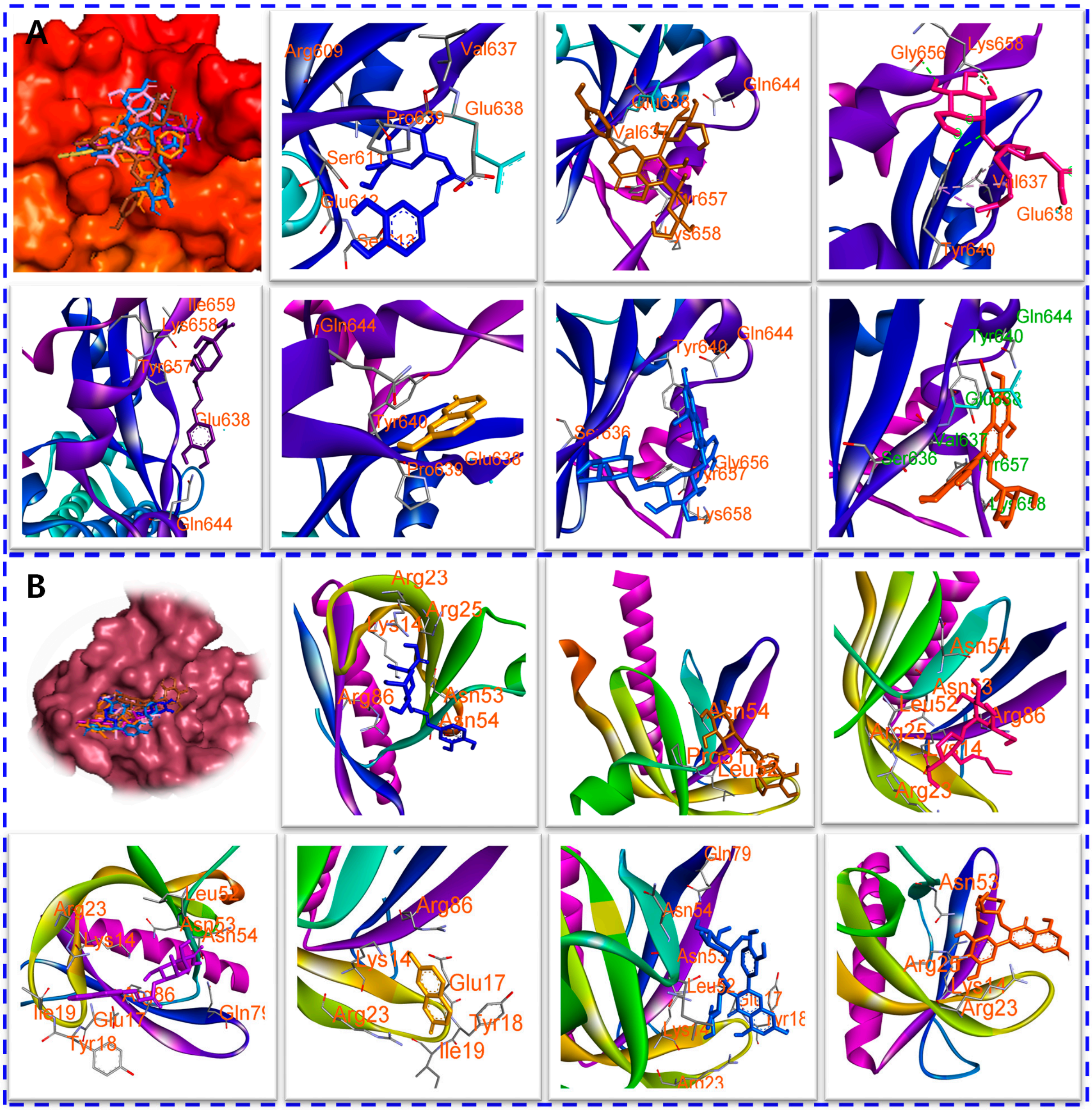

Figure S10. 3D Interactions of compounds, **9** (blue), **10** (brown), **13** (pink), **14** (purple), **15** (dark yellow), **23** (marine), **24** (orange) identified from *Z. piperitum* collected in SDM region, with amino acids. They were docked into STAT3 (A: 6NJS) and AKT1 (B: 1H10) proteins.

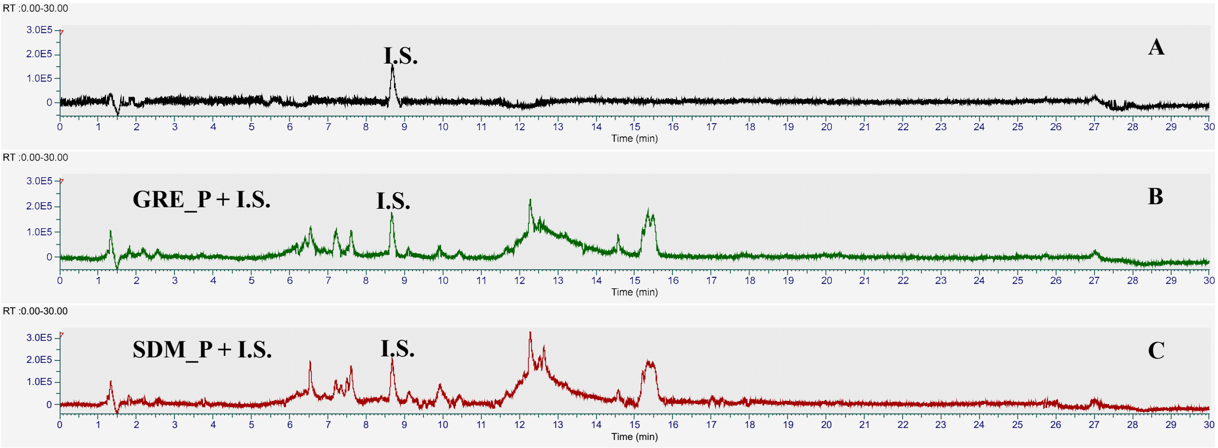

Figure S11. Chemical profiles of internal standard (A, caffeic acid: I.S.), and SDM (B) and GRE extracts spiked with internal standard (15 μg/mL).

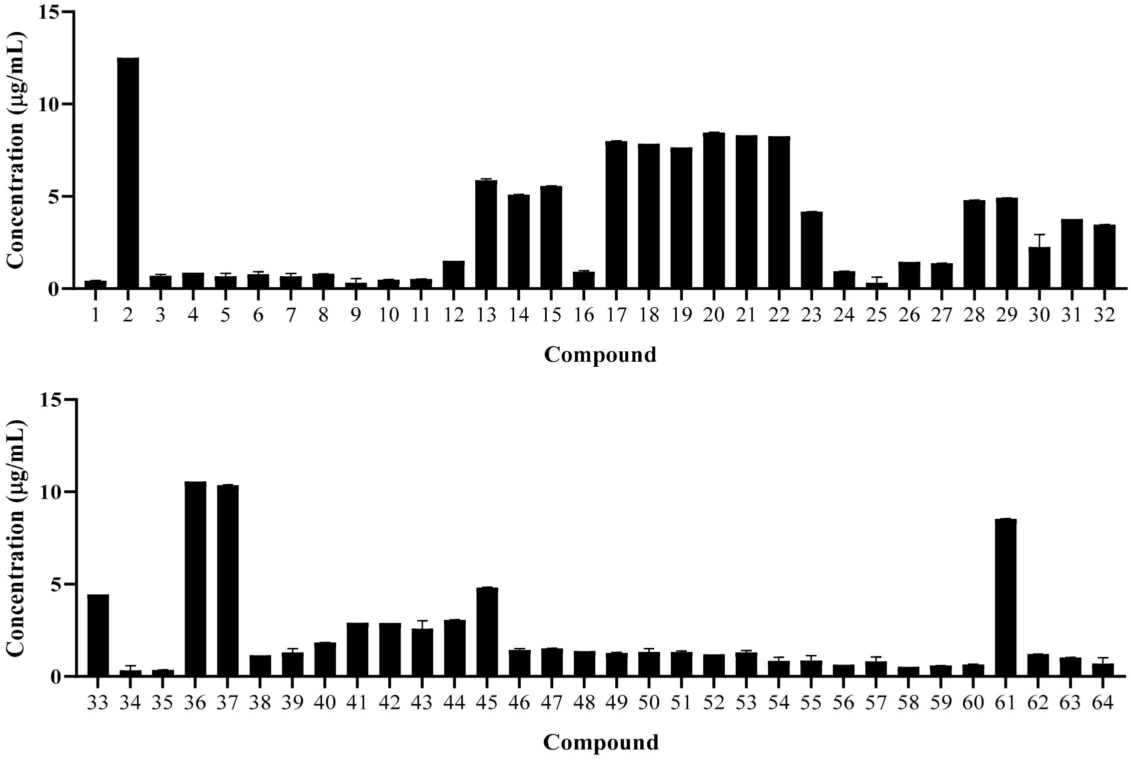

Figure S12. Relative quantities of compounds in relation to the internal standard detected from chromatogram extract of *Z. piperitum* collected at the GRE region.

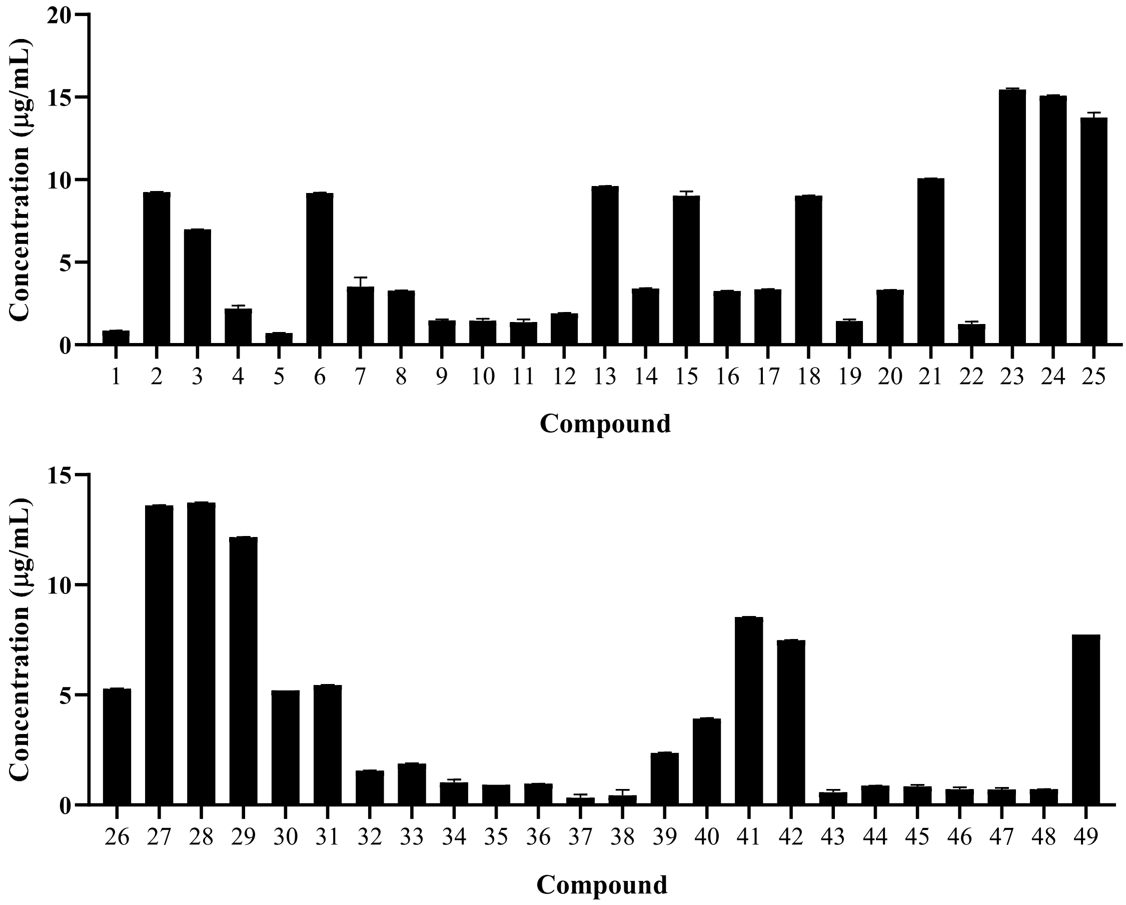

Figure S13. Relative quantities of compounds in relation to the internal standard detected from chromatogram extract of *Z. piperitum* collected at the SDM region.

Table S1. Extraction efficiency depends on extraction time.

Organ	Extraction Time (min)	Yield (mg)	SDM (%)	Yield (mg)	GRE (%)
F	0	0	0	0	0
	30	150.3	15.0	161.7	16.2
	60	175.2	17.5	166.4	16.6
	90	179.6	17.9	170.0	17.0
	120	206.0	20.6	172.8	17.3
	150	207.2	20.7	174.9	17.5
P	0	0	0	0	0
	30	280.8	28.1	203.1	20.3
	60	290.0	29.0	233.2	23.3
	90	290.9	29.1	234.8	23.5
	120	309.2	30.9	331.2	33.1
	150	310.6	31.0	333.3	33.3
S	0	0	0	0	0
	30	53.3	5.3	45.2	4.5
	60	90.7	9.1	47.9	4.8
	90	96.0	9.6	59.0	5.9
	120	130.9	13.1	75.7	7.6
	150	133.0	13.3	76.7	7.7

Table S2. Peak reduction of active compounds from LC-DPPH.

Region	No.	Compounds	Peak Reduction (%)
GRE	13	(3*S*)-3-(aminomethyl)-5-methylhexanoic acid	10.559
	17	4-Oxo-1*H*-quinoline-2-carboxylic acid	20.972
	18	2*E*-Hydroxypropane-1,2,3-tricarboxylic acid	20.972
	27	Dimethyl 2,4-bis(4-hydroxyphenyl) cyclobutane-1,3-dicarboxylate	29.888
	29	Unknown	28.35
	39	(2*S*,3*S*,4*S*,5*R*,6*S*)-3,4,5-trihydroxy-6-(4-methylphenoxy)oxane-2-carboxylic acid	29.842
	46	5,9-Dimethyltetracyclo[11.2.1.01,10.04,9]hexadecane-5,14-dicarboxylic acid	22.212
	61	3,3-Diethyl-5-methylpiperidine-2,4-dione	21.353
SDM	9	(1*S*,3*R*,4*R*,5*R*)-3-[(*E*)-3-(3,4-dihydroxyphenyl)prop-2-enoyl]oxy-1,4,5-trihydroxycyclohexane-1-carboxylic acid	22.926
	10	5,7-dihydroxy-2-(4-hydroxyphenyl)-6,8-bis[3,4,5-trihydroxy-6-(hydroxymethyl)oxan-2-yl]-2,3-dihydrochromen-4-one	22.926
	13	2-[2-[(*Z*)-pent-2-enyl]-3-[3,4,5-trihydroxy-6-(hydroxymethyl)oxan-2-yl]oxycyclopentyl]acetic acid	39.828
	14	*Trans*-(3*R*,5*R*)-4-[(*E*)-3-(3,4-dihydroxyphenyl)prop-2-enoyl]oxy-1,3,5-trihydroxycyclohexane-1-carboxylic acid	39.828
	15	7-Hydroxychromen-2-one	39.828
	23	2-(3,4-dihydroxyphenyl)-5,7-dihydroxy-3-[(2*S*,3*R*,4*S*,5*S*,6*R*)-3,4,5-trihydroxy-6-[[(2*R*,3*R*,4*R*,5*R*,6*S*)-3,4,5-trihydroxy-6-methyloxan-2-yl]oxymethyl]oxan-2-yl]oxychromen-4-one	20.851
	24	5,7-Dihydroxy-3-(3,4,5-trihydroxyoxan-2-yl)oxy-2-(3,4,5-trihydroxyphenyl)chromen-4-one	20.851

Table S3. Identified compounds detected from SDM region

Peak	Metabolites	RT (min)	MF	Adduct	*m/z*	Error (mDA)	Compound group
1	(2*S*)-2-amino-5-(diaminomethylideneamino)pentanoic acid	1.225	C6H14N4O2	[M+H]+	175.11767	1.290	Amino acids
2	L-2-pyrrolidinecarboxylic acid	1.346	C5H9NO2	[M+H]+	116.0697	0.905	Amino acids
3	(1*S*,2*R*,7*R*,10*R*,14*R*)-4-hydroxy-2,6,6,10,14-pentamethyl-11,13-dioxatetracyclo[8.8.0.0^{2,7}.0^{12,17}]octadeca-4,12(17)-diene-3,16-dione	1.399	C21H28O4	[M+Na]+	367.18422	3.769	Terpenoids
4	(3*R*,4*R*,5*S*,6*R*)-3-amino-6-(hydroxymethyl)oxane-2,4,5-triol	1.943	C6H13NO5	[M+H]+	180.0853	1.294	Glycosides
5	Unknown	3.274	-	[M+H]+	353.16874	-	-
6	*N*-[2-(5-hydroxy-1*H*-indol-3-yl)ethyl]acetamide	6.0933	C12H14N2O2	[M+H]+	219.1479	0.034	Phenols
7	2-(3,4-dihydroxyphenyl)-5-hydroxy-7-[(2*S*,3*R*,4*S*,5*S*,6*R*)-3,4,5-trihydroxy-6-[[(2*R*,3*R*,4*R*,5*R*,6*S*)-3,4,5-trihydroxy-6-methyloxan-2-yl]oxymethyl]oxan-2-yl]oxy-2,3-dihydrochromen-4-one	7.438	C27H32O15	[M-H]-	595.1649	1.907	Flavonoids
8	Unknown	7.453	_	[M+H]+	373.1815	-	_
9	(1*S*,3*R*,4*R*,5*R*)-3-[(*E*)-3-(3,4-dihydroxyphenyl)prop-2-enoyl]oxy-1,4,5-trihydroxycyclohexane-1-carboxylic acid	7.47	C16H18O9	[M-H]-	353.0865	1.298	Organic acids
10	5,7-dihydroxy-2-(4-hydroxyphenyl)-6,8-bis[3,4,5-trihydroxy-6-(hydroxymethyl)oxan-2-yl]-2,3-dihydrochromen-4-one	7.47	C27H32O15	[M+H]+	597.1787	2.671	Glycosides
11	(2*R*,3*S*,4*S*,4*aR*,10*bS*)-3,4,8,10-tetrahydroxy-2-(hydroxymethyl)-9-methoxy-3,4,4*a*,10*b*-tetrahydro-2H-pyrano[3,2-c]isochromen-6-one	7.537	C14H16O9	[M-H]-	327.0751	2.987	Benzopyrans
12	2-(3-methylbut-2-enyl)-5-(3-methylbut-3-en-1-ynyl)benzene-1,4-diol	7.542	C16H18O2	[M+NH_4]+	260.1624	2.015	Phenols
13	2-[2-[(*Z*)-pent-2-enyl]-3-[3,4,5-trihydroxy-6-(hydroxymethyl)oxan-2-yl]oxycyclopentyl]acetic acid	7.566	C18H30O8	[M+H]+	375.1969	4.382	Organic acids
14	*Trans*-(3*R*,5*R*)-4-[(*E*)-3-(3,4-dihydroxyphenyl)prop-2-enoyl]oxy-1,3,5-trihydroxycyclohexane-1-carboxylic acid	7.571	C16H18O9	[M-H]-	353.0865	1.243	Quinic acids
15	7-Hydroxychromen-2-one	7.585	C9H6O7	[M+H]+	163.038	0.970	Flavonoids
16	3,9,10-Trihydroxy-4-(methoxymethyl)-1,7-dimethyl-6-oxobenzo[b][1,4]benzodioxepine-2-carboxylic acid	7.585	C18H16O9	[M+H]+	377.0817	4.922	Phenols
17	1,3,5-Trihydroxy-4-[(*E*)-3-(3-hydroxy-4-methoxyphenyl)prop-2-enoyl]oxycyclohexane-1-carboxylic acid	7.651	C17H20O9	[M-H]-	367.1022	1.251	Quinic acids
18	7-Hydroxy-4-methylchromen-2-one	7.666	C10H8O3	[M+H]+	177.0534	1.23	Flavonoids
19	3-[6-[(2*S*,3*R*,4*S*,5*S*,6*R*)-3,4,5-trihydroxy-6-(hydroxymethyl)oxan-2-yl]oxy-1-benzofuran-5-yl]propanoic acid	7.672	C17H20O9	[M+Na]+	391.097	2.87	Glycosides
20	(7*S*,13*aS*)-3,10-dimethoxy-7-methyl-6,8,13,13*a*-tetrahydro-5*H*-isoquinolino[2,1-b]isoquinolin-7-ium-2,9-diol	9.2833	-	[M+H]+	342.1676	0.399	Alkaloids
21	(1*S*)-1-[(3-hydroxy-4-methoxyphenyl)methyl]-6-methoxy-2-methyl-3,4-dihydro-1*H*-isoquinolin-7-	10.02	C19H23NO4	[M+H]+	330.16805	0.001	Alkaloids

	ol						
22	3-Hydroxy-6-[[(*E*)-3-hydroxy-2,4-dimethylhept-4-enoyl]amino]-2,4-dimethyl-5-oxohexanoic acid	12.104	C17H29NO6	[M-H]-	342.1915	0.333	Organic acids
23	2-(3,4-Dihydroxyphenyl)-5,7-dihydroxy-3-[(2*S*,3*R*,4*S*,5*S*,6*R*)-3,4,5-trihydroxy-6-[[(2*R*,3*R*,4*R*,5*R*,6*S*)-3,4,5-trihydroxy-6-methyloxan-2-yl]oxymethyl]oxan-2-yl]oxychromen-4-one	12.164	C27H30O16	[M+H]+	611.1582	2.386	Flavonoids
24	5,7-Dihydroxy-3-(3,4,5-trihydroxyoxan-2-yl)oxy-2-(3,4,5-trihydroxyphenyl)chromen-4-one	12.175	C20H18O12	[M-H]-	449.071	1.543	Flavonoids
25	(13*aR*)-2,3,9,10-tetramethoxy-6,8,13,13*a*-tetrahydro-5*H*-isoquinolino[2,1-b]isoquinoline	12.209	C21H25NO4	[M+H]+	356.1834 2	2.184	Alkaloids
26	2,3,9,10-Tetramethoxy-6,8,13,13*a*-tetrahydro-5H-isoquinolino[2,1-*b*]isoquinoline	12.217	C21H25NO4	[M+H]+	356.1831	2.518	Alkaloids
27	5-[[6-[5,7-Dihydroxy-2-(4-hydroxyphenyl)-4-oxochromen-3-yl]oxy-3,4,5-trihydroxyoxan-2-yl]methoxy]-3-hydroxy-3-methyl-5-oxopentanoic acid	12.249	C27H28O15	[M-H]-	591.135	1.137	Flavonoids
28	2-(3,4-Dihydroxyphenyl)-5,7-dihydroxy-3-[(2*S*,3*R*,4*S*,5*R*,6*R*)-3,4,5-trihydroxy-6-(hydroxymethyl)oxan-2-yl]oxychromen-4-one	12.316	C21H20O12	[M+H]+	465.0994 9	0.003	Flavonoids
29	2-(3,4-Dihydroxyphenyl)-3,5,7-trihydroxychromen-4-one	12.322	C15H10O7	[M+H]+	303.0482 5	0.005	Flavonoids
30	(6*R*,6*aS*,8*R*,10*S*,10*aR*)-6,6*a*,8,10-tetramethyl-6,7,8,9,10,10a-hexahydro-2*H*-isochromeno[4,3-c]pyridin-1-one	12.334	C16H23NO2	[M+H]+	262.1784 4	1.701	Chromenopyridines
31	5,7-Dihydroxy-2-(4-hydroxyphenyl)-3-[(2*S*,3*R*,4*S*,5*S*,6*R*)-3,4,5-trihydroxy-6-(hydroxymethyl)oxan-2-yl]oxychromen-4-one	12.687	C21H20O11	[M+H]+	449.1055 4	0.005	Flavonoids
32	Methyl 2-[[3-[(3,3-dimethyloxiran-2-yl)methyl]-4-hydroxyphenyl]methyl]-4-hydroxy-3-(4-hydroxyphenyl)-5-oxofuran-2-carboxylate	13.011	C24H24O8	[M+H]+	441.1698	1.797	Fatty acids
33	2-[2-[(*Z*)-pent-2-enyl]-3-[3,4,5-trihydroxy-6-(hydroxymethyl)oxan-2-yl]oxycyclopentyl]acetic acid	13.042	C18H30O8	[M+HCOO]-	419.1907	1.492	Fatty acid
34	6-Methyl-9-azatricyclo[7.4.3.04,13]hexadec-1(13)-ene-2,8-dione	13.248	C16H23NO2	[M+H]+	262.1784 9	1.507	Alkaloids
35	(4*R*,4*aR*,7*S*,7*aR*,12*bS*)-9-methoxy-3-methyl-2,4,4*a*,5,6,7,7*a*,13-octahydro-1*H*-4,12-methanobenzofuro[3,2-e]isoquinolin-7-ol	13.248	C18H23NO3	[M+H]+	302.1713	3.812	Alkaloids
36	2-Heptylquinolin-4(1*H*)-one	13.25	C16H21NO	[M+H]+	244.1681	1.006	Quinolines
37	(1*R*,4*S*,5*R*,6*R*,8*S*,9*S*)-5,6,8-trihydroxy-6-methyl-13-azatetracyclo[7.7.0.01,13.04,9]hexadecan-2-one	13.983	C16H25NO4	[M+H]+	296.1841 1	1.674	Alkaloids
38	(6*R*,6*aS*,8*R*,10*S*,10*aR*)-6,6*a*,8,10-tetramethyl-6,7,8,9,10,10*a*-hexahydro-2*H*-isochromeno[4,3-c]pyridin-1-one	14.427	C16H23NO2	[M+H]+	262.1785	1.585	Alkaloids
39	Unknown	14.546	_	[M+H]+	302.1708	-	_
40	(1*S*,2*S*,4*R*,6*S*,9*S*)-2-hydroxy-4-methyl-13-azatetracyclo[7.7.0.01,6.02,13]hexadecan-8-one	15.422	C16H25NO2	[M+H]+	264.1943	1.491	Alkaloids
41	(2*E*,4*E*,8*Z*,10*E*,12*E*)-*N*-(2-hydroxy-2-methylpropyl)tetradeca-2,4,8,10,12-pentaenamide	17.094	C18H27NO2	[M+H]+	290.2097	1.023	Alkaloids
42	(2*E*,4*E*,8*E*,10*E*,12*E*)-*N*-(2-hydroxy-2-methylpropyl)tetradeca-2,4,8,10,12-	17.094	C18H27NO2	[M+H]+	290.2097	0.682	Alkaloids

	pentaenamide						
43	(2*S*)-1-[(2*R*)-2-methyldecanoyl]pyrrolidine-2-carboxylic acid	18.14		[M+Na]+	306.2041	0.096	Terpenoids
44	4-(2-Ethyl-6-methylphenyl)-5-methylmorpholin-3-one	19.074	C14H19NO2	[M+H]+	234.1474	1.51	Alkaloids
45	(4*S*,5*E*,6*S*)-4-(2-methoxy-2-oxoethyl)-5-[2-[(*E*)-3-phenylprop-2-enoyl]oxyethylidene]-6-[(2*S*,3*R*,4*S*,5*S*,6*R*)-3,4,5-trihydroxy-6-(hydroxymethyl)oxan-2-yl]oxy-4*H*-pyran-3-carboxylic acid	19.074	C14H25NO	[M+H]+	246.1838	1.527	Alkaloids
46	(2*E*,6*E*,8*E*,10*E*)-*N*-(2-methylpropyl)dodeca-2,6,8,10-tetraenamide	19.976	C16H25NO	[M+H]+	248.1989	0.729	Alkaloids
47	(2*E*,6*Z*,8*E*,10*E*)-*N*-(2,3-dihydroxy-2-methylpropyl)dodeca-2,6,8,10-tetraenamide	19.976	C16H25NO	[M+H]+	248.1989	0.618	Alkaloids
48	15-Methyl-6-azatetracyclo[8.6.0.01,6.02,13]hexadecan-11-one	19.976	C16H25NO	[M+H]+	248.1989	2.092	Alkaloids
49	(2*E*,4*E*)-*N*-(2-methylpropyl)dodeca-2,4-dienamide	24.064	C16H29NO	[M+Na]+	274.2147	0.008	Alkaloids

Table S4. Identified compounds detected from GRE region

Peak	Metabolites	RT (min)	MF	Adduct	*m/z*	Error (mDA)	Compound group
1	(2*S*)-2-amino-5-(diaminomethylideneamino)pentanoic acid	1.237	C6H14N4O2	[M+H]+	175.1176	1.362	Amino acids
2	3-Amino-2-methylpropanoic acid	1.309	C4H9NO2	[M+H]+	104.07	0.602	Amino acids
3	2-Hydroxypropane-1,2,3-tricarboxylic acid	2.131	C6H8O7	[M+Na]+	215.0146	2.131	Organic acids
4	(2*R*,3*R*,4*S*,5*R*)-2-(6-aminopurin-9-yl)-5-(hydroxymethyl)oxolane-3,4-diol	2.362	C10H13N5O4	[M+H]+	268.1025	2.362	Purine nucleosides
5	(4,7,7-trimethyl-3-bicyclo[2.2.1]heptanyl) (*E*)-3-(4-hydroxy-3-methoxyphenyl)prop-2-enoate	3.232	C20H26O4	[M+Na]+	353.16806	4.237	Organic acids
6	(1*S*,2*R*,7*R*,10*R*,14*R*)-4-hydroxy-2,6,6,10,14-pentamethyl-11,13-dioxatetracyclo[8.8.0.0^{2,7}.0^{12,17}]octadeca-4,12(17)-diene-3,16-dione	3.797	C21H28O4	[M+Na]+	367.18342	3.797	Terpenoids
7	2,3-Dihydro-1*H*-indole	5.816	C8H9N	[M+H]+	120.0800	0.74	Indoles
8	(2*S*)-2-amino-3-phenylpropanoic acid	5.816	C9H11NO2	[M+H]+	166.0850	1.177	Phenylalanine and derivatives
9	1-(1*H*-indol-3-yl)ethanone	6.077	C10H9NO	[M+H]+	160.0748	1.588	Alkaloids
10	(3*aR*,8*bS*)-3,4,8*b*-trimethyl-2,3*a*-dihydro-1*H*-pyrrolo[2,3-*b*]indol-7-ol	6.077	C13H18N2O	[M+H]+	219.1476	1.546	Alkaloids
11	3-[2-(Dimethylamino)ethyl]-1H-indol-5-ol	6.084	C12H16N2O	[M+H]+	205.1320	1.476	Alkaloids
12	Unknown	6.342	-	[M+Na]+	453.1839	-	-
13	(3*S*)-3-(aminomethyl)-5-methylhexanoic acid	6.478	C8H17NO2	[M+Na]+	182.1165	1.473	Organic acids
14	3-Acetyl-5-sec-butyl-4-hydroxy-1,5-dihydro-2*H*-pyrrol-2-one	6.567	C10H15NO3	[M+H]+	198.1112	1.247	Alkaloids
15	(*E*)-*N*-(4-aminobutyl)-3-(4-hydroxyphenyl)prop-2-enamide	6.698	C13H18N2O2	[M+H]+	235.1427	1.981	Alkaloids
16	(2*S*)-2-amino-3-(1*H*-indol-3-yl)propanoic acid	6.917	C11H12N2O2	[M+H]+	205.0959	1.195	Carboxylic acids
17	4-Oxo-1*H*-quinoline-2-carboxylic acid	7.138	C10H7NO3	[M+H]+	190.0488	1.066	Alkaloids
18	2*E*-hydroxypropane-1,2,3-tricarboxylic acid	7.143	C6H8O7	[M-H]-	191.0196	0.102	Organic acids
19	5,8-Dihydroxy-6-methoxy-7-[(2*S*,3*R*,4*S*,5*S*,6*R*)-3,4,5-trihydroxy-6-(hydroxymethyl)oxan-2-yl]oxychromen-2-one	7.196	C16H18O8	[M-H]-	385.0767	0.888	Phenols
20	7-Hydroxy-4-methylchromen-2-one	7.202	C10H8O3	[M+H]+	177.0535	1.068	Flavonoids
21	(1*R*,3*R*,4*S*,5*R*)-1,3,4-trihydroxy-5-[(*E*)-3-(4-hydroxyphenyl)prop-2-enoyl]oxycyclohexane-1-carboxylic acid	7.24	C16H18O8	[M-H]-	337.0926	0.206	Quinic acids
22	2,2'-[(4-Methylphenyl)imino]diethanol	7.246	C11H17NO2	[M+H]+	196.1320	1.160	Alkaloids
23	(*E*)-3-(3-hydroxy-4-methoxyphenyl)prop-2-enoic acid	7.593	C10H10O4	[M+H-H2O]+	177.0535	0.001	Phenols
24	(2*R*,3*S*,4*S*,5*R*,6*R*)-2-[[(2*R*,3*R*,4*R*)-3,4-dihydroxy-4-(hydroxymethyl)oxolan-2-yl]oxymethyl]-6-phenylmethoxyoxane-3,4,5-triol	7.886	C18H26O10	[M+NH4]+	420.1839	2.485	O-glycosyl compounds
25	3-Cyclohexyl-1,5,6,7-tetrahydrocyclopenta[d]pyrimidine-2,4-dione	8.711	C13H18N2O2	[M+H]+	235.1426	1.442	Alkaloids
26	(7*S*,13*aS*)-3,10-dimethoxy-7-methyl-6,8,13,13*a*-tetrahydro-5*H*-isoquinolino[2,1-b]isoquinolin-7-ium-2,9-diol	9.27	-	[M+H]+	342.1676	0.399	Alkaloids
27	Dimethyl 2,4-bis(4-hydroxyphenyl)cyclobutane-1,3-dicarboxylate	9.301	C20H20O6	[M+NH4]+	374.1578	2.012	Phenols
28	1-[(1*R*,2*R*)-2-hydroxy-2-(3-methoxyphenyl)cyclohexyl]-*N*,*N*-	9.814	C16H25NO3	[M+H]+	280.189	1.697	Alkaloids

	dimethylmethanamine oxide						
29	Unknown	9.88	_	[M+H]+	482.2327		_
30	(13*bS*)-2,11,12-trimethoxy-1,2,5,6,8,9-hexahydroindolo[7*a*,1-a]isoquinolin-3-one	10.013	C19H23NO4	[M+H]+	330.168	1.929	Alkaloids
31	3-[6-[(2*S*,3*R*,4*S*,5*S*,6*R*)-3,4,5-trihydroxy-6-(hydroxymethyl)oxan-2-yl]oxy-1-benzofuran-5-yl]propanoic acid	10.014	C17H20O9	[M+Na]+	391.0977	2.198	Glycosides
32	(1*S*)-1-[(3-hydroxy-4-methoxyphenyl)methyl]-6-methoxy-2-methyl-3,4-dihydro-1*H*-isoquinolin-7-ol	10.02	C19H23NO4	[M+H]+	330.1683	2.043	Alkaloids
33	(2*S*)-2-[[(3*aS*,6*R*,7*aS*)-6-ethyl-1-oxo-2,3,3*a*,6,7,7*a*-hexahydroindene-4-carbonyl]amino]-3-methylpentanoic acid	10.109	C18H27NO4	[M+H]+	320.1815	4.486	Alkaloids
34	(6*R*,6*aS*,8*R*,10*S*,10*aR*)-2-hydroxy-6,6a,8,10-tetramethyl-6,7,8,9,10,10a-hexahydroisochromeno[4,3-c]pyridin-1-one	11.793	C16H23NO3	[M+H]+	278.17344	1.643	Chromenopyridines
35	2-Methoxy-5-methyl-3-(2-methylbut-3-en-2-yl)chromen-4-one	11.801	C16H18O3	[M+NH4]+	276.1577	1.713	Aromatics
36	(2*S*,10*S*,13*S*,15*R*)-2-hydroxy-15-methyl-6-oxido-6-azoniatetracyclo[8.6.0.01,6.02,13]hexadecan-11-one)	12.1	C16H25NO3	[M+H]+	280.1889	1.008	Alkaloids
37	2-(3,4-dihydroxyphenyl)-5,7-dihydroxy-3-[(2*S*,3*R*,4*S*,5*R*,6*R*)-3,4,5-trihydroxy-6-(hydroxymethyl)oxan-2-yl]oxychromen-4-one	12.3164	C21H20O12	[M+H]+	465.0997	0.0032959	Flavonoids
38	Methyl 2-[[3-[(3,3-dimethyloxiran-2-yl)methyl]-4-hydroxyphenyl]methyl]-4-hydroxy-3-(4-hydroxyphenyl)-5-oxofuran-2-carboxylate	13.002	C24H24O8	[M+H]+	441.1695	2.631	Phenols
39	(2*S*,3*S*,4*S*,5*R*,6*S*)-3,4,5-trihydroxy-6-(4-methylphenoxy)oxane-2-carboxylic acid	13.158	C13H16O7	[M+NH4]+	302.1271	3.704	Glycosides
40	6-Methyl-9-azatricyclo[7.4.3.04,13]hexadec-1(13)-ene-2,8-dione	13.248	C16H23NO2	[M+H]+	262.1784	1.507	Alkaloids
41	2-Heptylquinolin-4(1*H*)-one	13.25	C16H21NO	[M+H]+	244.1681	1.006	Quinolines
42	(4*R*,4*aR*,7*S*,7*aR*,12*bS*)-9-methoxy-3-methyl-2,4,4*a*,5,6,7,7*a*,13-octahydro-1H-4,12-methanobenzofuro[3,2-e]isoquinolin-7-ol	13.455	C18H23NO3	[M+Na]+	302.1711	3.936	Alkaloids
43	4-(2-Ethyl-6-methylphenyl)-5-methylmorpholin-3-one	13.457	C14H19NO2	[M+H]+	234.1474	1.423	Alkaloids
44	(1*R*,4*S*,5*R*,6*R*,8*S*,9*S*)-5,6,8-trihydroxy-6-methyl-13-azatetracyclo[7.7.0.01,13.04,9]hexadecan-2-one	13.983	C16H25NO4	[M+H]+	296.1841	1.6747	Alkaloids
45	(1*S*,2*S*,4*R*,6*S*,9*S*)-2-hydroxy-4-methyl-13-azatetracyclo[7.7.0.01,6.02,13]hexadecan-8-one	15.422	C16H25NO2	[M+H]+	264.1943	1.491	Alkaloids
46	5,9-Dimethyltetracyclo[11.2.1.01,10.04,9]hexadecane-5,14-dicarboxylic acid	16.934	C20H30O4	[M+H]+	335.2168	4.882	Terpenoids
47	(3*S*,5*S*,8*R*,9*S*,10*S*,13*R*,14*S*,17*R*)-5,14-dihydroxy-13-methyl-17-(6-oxopyran-3-yl)-3-[(2*R*,3*R*,4*S*,5*S*,6*R*)-3,4,5-trihydroxy-6-(hydroxymethyl)oxan-2-yl]oxy-2,3,4,6,7,8,9,11,12,15,16,17-dodecahydro-1*H*-cyclopenta[a]phenanthrene-10-carbaldehyde	17.016	C30H42O11	[M+NH4]+	596.306	0.466	Glycosides
48	(2*E*,4*E*,8*Z*,10*E*,12*E*)-*N*-(2-hydroxy-2-methylpropyl)tetradeca-2,4,8,10,12-pentaenamide	17.094	C18H27NO2	[M+H]+	290.2097	0.237	Alkaloids

49	(2*E*,4*E*,8*E*,10*E*,12*E*)-*N*-(2-hydroxy-2-methylpropyl)tetradeca-2,4,8,10,12-pentaenamide	17.094	C18H27NO2	[M+H]+	290.2097	0.42	Alkaloids
50	Unknown	17.815	C17H3N9O	[M-H]-	348.0379	-	_
51	4-Hydroxy-2-methoxy-6-methylbenzoic acid	17.997	C9H10O4	[M+Cl]-	217.0265	0.48	Organic acids
52	(*E*)-*N*-[(4-hydroxy-3-methoxyphenyl)methyl]-8-methylnon-6-enamide	18.001	C18H27NO3	[M+H]+	306.2046	1.696	Phenols
53	(4*Z*,7*Z*,10*Z*,13*Z*)-*N*-(2-hydroxyethyl)hexadeca-4,7,10,13-tetraenamide	18.04	C18H29NO2	[M+H]+	292.22521	1.905	Alkaloids
54	(2*S*)-1-[(2*R*)-2-methyldecanoyl]pyrrolidine-2-carboxylic acid	18.14	C16H29NO3	[M+Na]+	306.2041	0.096	Alkaloids
55	(4*S*,5*E*,6*S*)-4-(2-methoxy-2-oxoethyl)-5-[2-[(*E*)-3-phenylprop-2-enoyl]oxyethylidene]-6-[(2*S*,3*R*,4*S*,5*S*,6*R*)-3,4,5-trihydroxy-6-(hydroxymethyl)oxan-2-yl]oxy-4*H*-pyran-3-carboxylic acid	19.071	C14H25NO	[M+Na]+	246.1837	1.472	Alkaloids
56	4-(2-Ethyl-6-methylphenyl)-5-methylmorpholin-3-one	19.098	C14H19NO2	[M+H]+	234.1470	1.851	Alkaloids
57	*N*-(9-oxodecyl)acetamide	19.666	C12H23NO2	[M+Na]+	236.1628	0.736	Alkaloids
58	(2*E*,6*E*,8*E*,10*E*)-*N*-(2-methylpropyl)dodeca-2,6,8,10-tetraenamide	19.976	C16H25NO	[M+H]+	248.1989	0.729	Alkaloids
59	(2*E*,6*Z*,8*E*,10*E*)-*N*-(2,3-dihydroxy-2-methylpropyl)dodeca-2,6,8,10-tetraenamide	19.976	C16H25NO	[M+H]+	248.1989	0.618	Alkaloids
60	15-Methyl-6-azatetracyclo[8.6.0.01,6.02,13]hexadecan-11-one	19.976	C16H25NO	[M+H]+	248.1989	2.092	Alkaloids
61	3,3-Diethyl-5-methylpiperidine-2,4-dione	20.16	C10H17NO2	[M+H]+	184.1319	1.154	Alkaloids
62	(1*S*,4*S*,7*S*,8*R*,11*R*,12*R*,13*S*)-2,12-dimethyl-13-propan-2-yl-10-oxa-2-azatetracyclo[5.4.1.18,11.04,12]tridecan-9-one	20.317	C16H25NO2	[M+H]+	264.1942	2.195	Alkaloids
63	*N*-(9*S*-oxodecyl)acetamide	20.321	C12H23NO2	[M+Na]+	236.1631	1.095	Alkaloids
64	(2*E*,4*E*)-*N*-(2-methylpropyl)dodeca-2,4-dienamide	24.085	C16H29NO	[M+Na]+	274.2146	0.008	Alkaloids

Table S5. Quantitation of identified compounds from *Z. piperitum* collected in the GRE region.

Compound	RPA (%)	Amount (μg/mL)	Component
1	2.80 ± 0.11	0.42 ± 0.02	Trace
2	83.41 ± 0.08	12.51 ± 0.01	Major
3	4.61 ± 0.37	0.69 ± 0.06	Minor
4	5.80 ± 0.02	0.87 ± 0.00	Minor
5	4.50 ± 0.91	0.67 ± 0.14	Minor
6	5.25 ± 0.71	0.79 ± 0.11	Minor
7	4.46 ± 0.81	0.67 ± 0.12	Minor
8	5.31 ± 0.04	0.80 ± 0.01	Minor
9	2.10 ± 1.26	0.31 ± 0.19	Trace
10	3.26 ± 0.05	0.49 ±0.01	Trace
11	3.44 ± 0.01	0.52 ± 0.00	Minor
12	9.98 ± 0.01	1.50 ± 0.00	Minor
13	39.17 ± 0.39	5.87 ± 0.06	Major
14	33.93 ± 0.09	5.09 ± 0.01	Major
15	37.04 ± 0.13	5.56 ± 0.02	Major
16	6.07 ± 0.33	0.91 ± 0.05	Major
17	53.24 ± 0.15	7.99± 0.02	Major
18	52.32 ± 0.07	7.85 ± 0.01	Major
19	50.97 ± 0.02	7.64 ± 0.00	Major
20	56.38 ± 0.10	8.46± 0.02	Major
21	55.38 ± 0.03	8.31 ± 0.00	Major
22	55.08 ± 0.02	8.26 ± 0.00	Major
23	27.78 ± 0.05	4.17 ± 0.01	Minor
24	6.27 ± 0.05	0.94 ±0.01	Minor
25	2.09 ± 1.66	0.31 ± 0.25	Trace
26	9.64 ± 0.02	1.45 ± 0.00	Minor
27	9.17 ± 0.00	1.38 ± 0.00	Minor
28	31.91 ± 0.06	4.79 ± 0.01	Minor
29	32.77 ± 0.02	4.92 ± 0.00	Minor
30	15.08 ± 3.63	2.26 ± 0.54	Minor
31	25.12 ± 0.01	3.77 ± 0.00	Minor
32	23.09 ± 0.03	3.46 ± 0.00	Minor
33	29.61 ± 0.01	4.44 ± 0.00	Minor
34	2.23 ± 1.33	0.33 ± 0.20	Trace
35	2.31 ± 0.01	0.35 ± 0.00	Trace
36	70.45 ± 0.04	10.57 ± 0.01	Major
37	69.09 ± 0.09	10.36 ± 0.01	Major
38	7.58 ± 0.00	1.14 ± 0.00	Minor
39	8.74 ± 1.02	1.31 ± 0.15	Minor
40	12.24 ± 0.00	1.84 ± 0.00	Minor
41	19.36 ± 0.02	2.90 ± 0.00	Minor
42	19.28 ± 0.03	2.89 ± 0.00	Minor
43	17.19 ± 2.41	2.58 ± 0.36	Minor

**Table S5.** Continue			
44	20.35 ± 0.08	3.05 ± 0.01	Minor
45	32.06 ±0.09	4.81 ± 0.01	Minor
46	9.56 ± 0.43	1.43 ± 0.06	Minor
47	10.16 ± 0.05	1.52 ± 0.01	Minor
48	9.16 ± 0.00	1.37 ± 0.00	Minor
49	8.47 ± 0.19	1.27 ± 0.03	Minor
50	8.90 ± 0.91	1.34 ± 0.14	Minor
51	8.81 ± 0.34	1.32 ± 0.05	Minor
52	7.94 ± 0.05	1.19 ± 0.001	Minor
53	8.69 ± 0.59	1.30 ± 0.09	Minor
54	5.65 ± 1.11	0.85 ± 0.17	Minor
55	5.73 ± 1.47	0.86 ± 0.22	Minor
56	4.22 ± 0.00	0.63 ± 0.00	Minor
57	5.47 ± 1.29	0.82 ±0.19	Minor
58	3.46 ± 0.01	0.52 ±0.00	Minor
59	3.94 ± 0.04	0.59 ± 0.01	Minor
60	4.28 ± 0.19	0.64 ± 0.03	Minor
61	56.92 ± 0.04	8.54 ± 0.01	Major
62	8.08 ± 0.06	1.21 ± 0.01	Minor
63	6.79 ± 0.06	1.02 ± 0.01	Minor
64	4.61 ± 1.80	0.69 ± 0.27	Minor

RPA is relative peak area (%) and was calculated by using formula:

RPA= Compound peak area * 100/ IS peak area

Amount (µg/mL) = Compound peak area * 15/ IS peak area

Classification of compounds based on their amount (µg/mL) by using the following criteria:
major (Amount ≥ 5); minor (5.0 > Amount ≥ 0.5); trace (Amount < 0.5).

Table S6. Quantitation of identified compounds from *Z. piperitum* collected in the SDM region.

Compound	RPA (%)	Amount (μg/mL)	Component
1	5.67 ± 0.04	0.85 ± 01	Minor
2	61.56 ± 0.11	9.23 ± 0.02	Major
3	46.58 ± 0.04	6.99 ± 0.01	Major
4	14.64 ± 0.91	2.20 ± 0.14	Minor
5	4.67 ± 0.17	0.70 ± 0.03	Minor
6	61.32 ± 0.03	9.20 ± 0.00	Major
7	23.43 ± 3.04	3.51 ± 0.46	Minor
8	21.85 ± 0.05	3.28 ± 0.01	Minor
9	9.82 ± 0.42	1.47 ± 0.06	Minor
10	9.66 ± 0.67	1.45 ± 0.10	Minor
11	9.16 ± 0.93	1.37 ± 0.14	Minor
12	12.74 ± 0.07	1.91 ± 0.01	Minor
13	64.00 ± 0.06	9.60 ± 0.01	Major
14	22.77 ± 0.05	3.42 ± 0.01	Minor
15	60.18 ± 1.44	9.03 ± 0.22	Major
16	21.76 ± 0.06	3.26 ± 0.01	Minor
17	22.40 ± 0.03	3.36 ± 0.00	Minor
18	60.17 ± 0.07	9.03 ± 0.01	Major
19	9.66 ± 0.54	1.45 ± 0.08	Minor
20	22.12 ± 0.04	3.32 ± 0.01	Minor
21	67.13 ± 0.07	10.07 ± 0.01	Major
22	8.41 ± 0.82	1.26 ± 0.12	Minor
23	103.09 ± 0.32	15.46 ± 0.05	Major
24	100.45 ± 0.22	15.07 ± 0.03	Major
25	91.66 ± 1.67	13.75 ± 0.25	Major
26	35.18 ± 0.06	5.28 ± 0.01	Major
27	90.65 ± 0.10	13.60 ± 0.02	Major
28	91.50 ± 0.06	13.72 ± 0.01	Major
29	81.04 ± 0.09	12.16 ± 0.01	Major
30	34.72 ± 0.03	5.21 ± 0.00	Major
31	36.24 ± 0.09	5.44 ± 0.01	Major
32	10.39 ± 0.06	1.56 ± 0.01	Minor
33	12.59 ± 0.04	1.89 ± 0.01	Minor
34	6.93 ± 0.63	1.04 ± 0.09	Minor
35	6.13 ± 0.01	0.92 ± 0.00	Minor
36	6.41 ± 0.05	0.96 ± 0.01	Minor
37	2.23 ± 0.83	0.33 ± 0.12	Trace
38	2.99 ± 1.38	0.45 ± 0.21	Trace
39	15.81 ± 0.04	2.37 ± 0.01	Minor
40	26.17 ± 0.06	3.92 ± 0.01	Minor
41	56.87 ± 0.08	8.53 ± 0.01	Major
42	49.86 ± 0.04	7.48 ± 0.01	Major
43	3.86 ± 0.59	0.58 ± 0.09	Minor

**Table S6.** Continue			
44	5.85 ± 0.04	0.88 ± 0.01	Minor
45	5.62 ± 0.37	0.84 ± 0.06	Minor
46	4.77 ± 0.49	0.72 ± 0.07	Minor
47	4.72 ± 0.39	0.71 ± 0.06	Minor
48	4.76 ± 0.04	0.71 ± 0.01	Minor
49	51.61 ± 0.03	7.74 ± 0.01	Major

RPA is relative peak area (%) and was calculated by using formula:

RPA= Compound peak area * 100/ IS peak area

Amount/ IS (µg/mL) = Compound peak area * 15/ IS peak area

Classification of compounds based on their amount (µg/mL) by using the following criteria:

major (Amount ≥ 5); minor (5.0 > Amount ≥ 0.5); trace (Amount < 0.5).
